# Filtration of Macrophage Migration Inhibitory Factor (MIF) in Patients with End Stage Renal Disease Undergoing Hemodialysis

**DOI:** 10.1371/journal.pone.0140215

**Published:** 2015-10-20

**Authors:** Peter Luedike, Christos Rammos, Julia Pohl, Martin Heisler, Matthias Totzeck, Werner Kleophas, Gerd R. Hetzel, Malte Kelm, Ulrike Hendgen-Cotta, Tienush Rassaf

**Affiliations:** 1 University Hospital Düsseldorf, Medical Faculty, Division of Cardiology, Pulmonology and Vascular Medicine, Moorenstrasse 5, 40225 Düsseldorf, Germany; 2 DaVita Renal Center, Bismarckstraße 101, 40210 Düsseldorf, Germany; KRH Robert Koch Klinikum Gehrden, GERMANY

## Abstract

**Background:**

End stage renal disease (ESRD) patients are characterized by increased morbidity and mortality due to highest prevalence of cardiovascular disease. Macrophage migration inhibitory factor (MIF) is an inflammatory cytokine that controls cellular signaling in human physiology, pathophysiology, and diseases. Increased MIF plasma levels promote vascular inflammation and development of atherosclerosis. We have shown that MIF is associated with vascular dysfunction in ESRD patients. Whether hemodialysis (HD) affects circulating MIF plasma levels is unknown. We here aimed to investigate whether HD influences the circulating MIF pool in ESRD patients.

**Methods and Results:**

An observational single-center study was conducted. MIF plasma levels in ESRD patients were assessed before, during, and after a HD session (n = 29). Healthy age-matched volunteers served as controls to compare correlations of MIF plasma levels with inflammatory plasma components (n = 20). MIF removed from the circulating blood pool could be detected in the dialysate and allowed for calculation of totally removed MIF (MIF content in dialysate 219±4 μg/HD-session). MIF plasma levels were markedly decreased 2 hour after initiation of HD (MIF plasma level pre-HD 84.8±6 ng/ml to intra-HD 61.2±5 ng/ml p<0.001) and were replenished already 20 min after termination of HD to basal levels (intra-HD 61.2±5 ng/ml to post-HD 79.8±5 ng/ml, p<0.001).

**Conclusion:**

MIF is a dialyzable plasma component that is effectively filtrated during HD from the patient blood pool in large amounts. After removal of remarkable amounts of MIF during a single HD session, MIF plasma pool is early reconstituted after termination of HD from unknown sources.

## Introduction

Patients with end stage renal disease (ESRD) are at the highest risk for the development of cardiovascular events like stroke or myocardial infarction and thus suffer from increased morbidity and mortality.[1–3] Sustained vascular inflammation is the cornerstone of increased cardiovascular risk in ESRD patients.[4], Hemodialysis (HD) per se influences inflammatory processes in patients under maintenance HD.[5] This influences vascular functions, which are blunted in ESRD and moreover after HD.[6, 7] In patients with ESRD, MIF plasma levels are associated with diminished endothelial function and increased vascular stiffness and correlate with myocardial injury.[8] ESRD goes along with systemic inflammation and is associated with increased platelet activation.[9] The impact of HD therapy on circulating MIF levels in ESRD patients has not been investigated so far.

MIF is a structurally unique inflammatory cytokine that controls cellular signaling in human physiology and disease.[10] It is critically involved in myocardial infarction and exhibits both intracellular properties as well as extracellular, receptor mediated chemokine functions.[11–13] Besides acting as an inflammatory cytokine, MIF also exhibits chemokine like functions and was found to bind to the chemokine receptors CXCR2 and CXCR4 and trigger leukocyte recruitment. MIF has gained attention due to its pro-atherogenic properties[12] and is secreted from immune cells upon diverse inflammatory stimuli like LPS, TNF-α or IFN-γ[14]. Non immune cells like endothelial cells, smooth muscle cells or parenchymal cells also secrete MIF after inflammatory or atherogenic stimulation with hypoxia, ROS or oxLDL[15]. MIF is also expressed in human and murine platelets and is secreted from platelets upon activation.[16] MIF was shown to be involved in inflammatory atherosclerosis pathogenesis and is implicated in modulation of disease progression.[15, 17] Plaque regression and a more stable plaque phenotype have been shown through MIF blockade in a pre-clinical setting.[12] Whilst MIF is in spotlight of experimental atherosclerosis research, clinical studies investigating MIFs impact are rare to date.

With regard to increasing amounts of literature on the role of MIF in cardiovascular diseases and the increased cardiovascular risk in ESRD, we here aimed to investigate the impact of HD on MIF plasma levels in ESRD patients.

## Patients and Methods

### Study population

**Fig 1:**
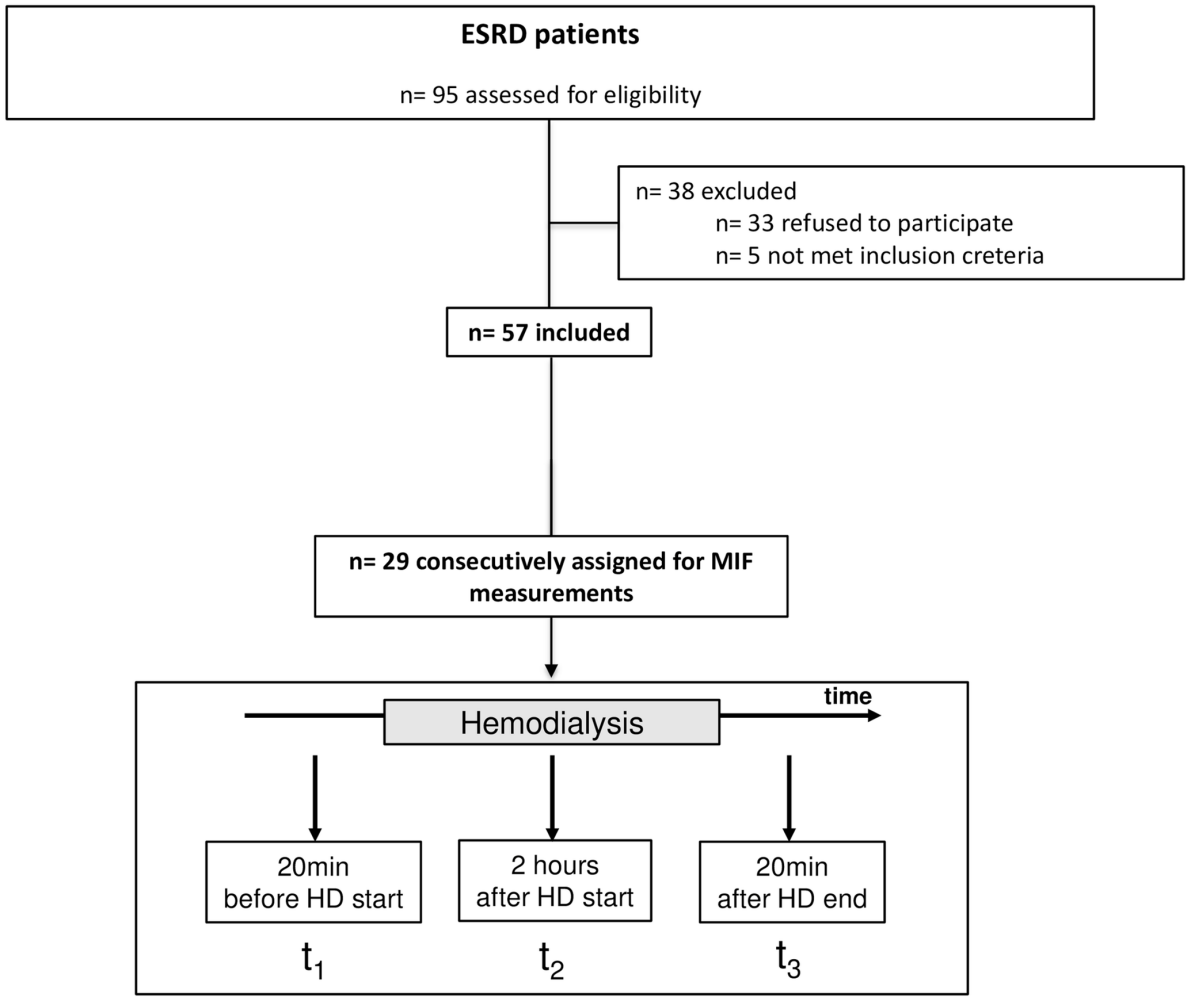
Flowchart of recruited patients. The included ESRD patients represent a random sample of a larger group of patients (n = 57) in which the influence of flavanols on vascular function in ESRD patients is investigated. Of these, n = 29 were consecutively assigned for analysis of MIF plasma level before, during and after hemodialysis (HD). The different time points for blood sample collection were 20 min before start of HD (t_1_), 2 hours after start of HD (t_2_) and 20 min after termination of HD (t_3_).

The study was registered at ClinicalTrials.gov (NCT number: NCT01412320) and the protocol for this trial is available as supporting information (see S1 and S2 Protocols). The included n = 29 ESRD patients represent a random sample of a larger group of patients (n = 57) in which the influence of flavanols on vascular function in ESRD patients is investigated. The investigated patients were consecutively assigned to the current study before they were randomized to the favanols trial between 07/2011 and 08/2011 in the DaVita Renal Center Duesseldorf (Fig 1). Study procedures were in accordance with the Declaration of Helsinki and the institutional Ethics Committee of the Heinrich-Heine University approved the study protocol (No. 3587). N = 29 patients with ESRD undergoing maintenance hemodialysis gave written informed consent and were included in this study (Table 1). N = 28 patients were found to be not eligible due to missing blood samples after HD. Healthy age-matched volunteers served as controls (n = 20). Due to the known circadian rhythm of MIF, examinations were conducted and blood samples were taken before 1:00 pm.[18] Blood was drawn following a 15-minute rest for clinical routine and inflammatory parameters (e.g. MIF). The different time points for blood sample collection were 20 min before start of HD (t_1_), 2 hours after start of HD (t_2_) and 20 min after termination of HD (t_3_) (Fig 1). The Institute of Clinical Chemistry and Laboratory Diagnostic, University Hospital Duesseldorf performed all analyses unless noted otherwise. Markers of oxidative stress oxidized LDL (OxLDL, Mercodia, Uppsala, Sweden) were measured by ELISAs following the manufacturers protocol.

**Table 1:** Basic clinical and biochemical characteristics.

		**ESRD**
n		29
Age (y)		66 ± 13
Male gender (n)		22
Height (cm)		174 ±10
Weight (kg)		86 ± 19
Renal diagnosis	Hypertensive/large vessel	12
	Diabetic nephropathy	5
	Glomerulonephritis	5
	Polycystic kidney disease	4
	Other/miscellaneous	3
Dialysis vintage (months)		50±35
Cardiovascular disease (n)		12
Risk factors (n)	Hypertension	26
	Diabetes	9
	Current smoker	8
	Hypercholesterolemia	14
Medication (n)	ASS	16
	Statin	14
	AT blocker	
	ACE-I	6
	Beta blocker	16
	Ca channel blocker	12
	Diuretics	21
	Steroids	2
Chemistry Panel	Sodium (mmol/l)	140.7 ± 2.5
	Potassium (mmol/l)	4.9 ± 0.9
	Calcium (mmol/l)	2.2 ± 0.2
	C-reactive protein (mg/dl)	0.8 ± 1.7
	Total protein (g/dl)	6.9 ± 0.5
	Cystatin C (mg/dl)	5.7 ± 1.2
	Creatinine (mg/dl)	8.2 ± 2.5
	Urea nitrogen (mg/dl)	121 ± 34
	Total cholesterol (mg)	175 ± 38
	HS-troponin (ng/l)	59 ± 38
	Creatin-kinase (mg/dl)	81.4 ± 43.7
Blood Count	Hemoglobin (g/dl)	11.2 ± 0.8
	Hematocrit (%)	35.2 ± 2.7
	Platelets (/ll)	217.0 ± 57.5
	Leucocytes (104/ll)	7.5 ± 2.3
		**ESRD**
n		29
Age (y)		66 ± 13
Male gender (n)		22
Height (cm)		174 ±10
Weight (kg)		86 ± 19
Renal diagnosis	Hypertensive/large vessel	12
	Diabetic nephropathy	5
	Glomerulonephritis	5
	Polycystic kidney disease	4
	Other/miscellaneous	3
Dialysis vintage (months)		50±35
Cardiovascular disease (n)		12
Risk factors (n)	Hypertension	26
	Diabetes	9
	Current smoker	8
	Hypercholesterolemia	14
Medication (n)	ASS	16
	Statin	14
	AT blocker	
	ACE-I	6
	Beta blocker	16
	Ca channel blocker	12
	Diuretics	21
	Steroids	2
Chemistry Panel	Sodium (mmol/l)	140.7 ± 2.5
	Potassium (mmol/l)	4.9 ± 0.9
	Calcium (mmol/l)	2.2 ± 0.2
	C-reactive protein (mg/dl)	0.8 ± 1.7
	Total protein (g/dl)	6.9 ± 0.5
	Cystatin C (mg/dl)	5.7 ± 1.2
	Creatinine (mg/dl)	8.2 ± 2.5
	Urea nitrogen (mg/dl)	121 ± 34
	Total cholesterol (mg)	175 ± 38
	HS-troponin (ng/l)	59 ± 38
	Creatin-kinase (mg/dl)	81.4 ± 43.7
Blood Count	Hemoglobin (g/dl)	11.2 ± 0.8
	Hematocrit (%)	35.2 ± 2.7
	Platelets (/ll)	217.0 ± 57.5
	Leucocytes (104/ll)	7.5 ± 2.3

### MIF levels

MIF was determined as described previously.[19, 20] Heparinized full blood was centrifuged at 800 g for 10 min (4°Celsius). The resulting plasma aliquots were snap frozen in liquid nitrogen and stored at -80°Celsius until further analysis.

For the determination of MIF in the dialysate ultrafiltrate samples were taken from the Genius dialysis system 30 minutes after initiation and 30 minutes before end of the HD-session, respectively.

MIF levels were measured by quantitative sandwich enzyme-linked immunosorbent assay (ELISA) (Quantikine, R&D Systems, Minneapolis, USA) according to the manufacturer’s protocols.

### Genius dialysis system

The GENIUS hemodialysis system is a standardized, industrial dialysis device (Fresenius^®^).[21] For each treatment, fresh dialysis fluid is prepared according to the physician's prescription with preheated ultrapure water. The total amount of dialysis fluid is put into a thermally insulated glass tank (volume 70 l) of the hemodialysis machine using an UV radiator used for disinfection. During treatment, fresh dialysis fluid is taken from the top of the system, and the used dialysate is returned to the bottom. There is a sharp interface between the fresh and used dialysis fluids because of a small difference in temperature.[21] Ultrafiltration rates between 100 and 1,000 ml/h can be selected as considered appropriate by the physician.

### Statistical methods

Results are expressed as mean ± standard deviation (SD) unless stated otherwise. All data were checked for normality distribution using Kolmogorov-Smirnov test and no departures were noted. Differences between groups were compared using unpaired Student’s two-tailed t-test. Correlations between individual parameters were calculated using univariate analyses. Results are expressed as Pearson’s r and corresponding p values. P values of less than 0.05 were regarded statistically significant. All statistical tests were conducted using Prism 5.0 (GraphPad) for Mac OS.

## Results

### MIF is a dialyzable plasma component that is effectively removed from blood during HD

**Fig 2:**
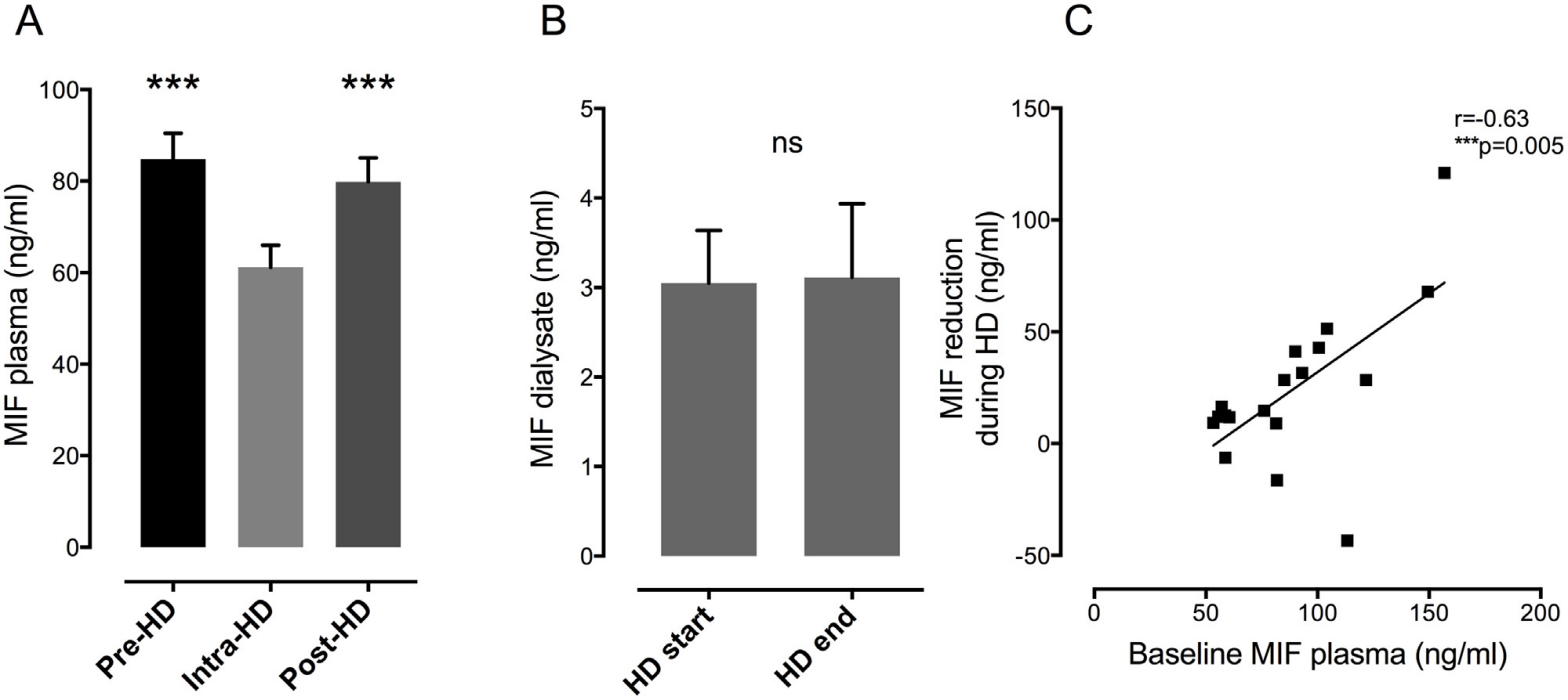
MIF plasma level decrease during HD and restore after termination of renal replacement therapy. **A**) MIF plasma level decrease during HD (Intra-HD) compared to baseline levels (Pre-HD) (p<0.01). After termination of HD (Post-HD), MIF plasma level return to baseline values (Pre-HD vs. Post-HD; p = ns). **B**) MIF can be measured in the ultrafiltrate (UF) immediately after beginning of HD (HD start). Concentration of MIF in UF does not change over time as demonstrated by measurements at the end of HD session (HD end)(p = ns). **C**) The absolute amount of removed MIF is associated with the baseline amounts of MIF plasma levels in ESRD patients (p<0.01).

Since patients with ESRD are characterized by the highest cardiovascular disease risk, we assessed MIF plasma levels in patients with ESRD undergoing maintenance HD before, during and after a HD session (Fig 2).

**Fig 3:**
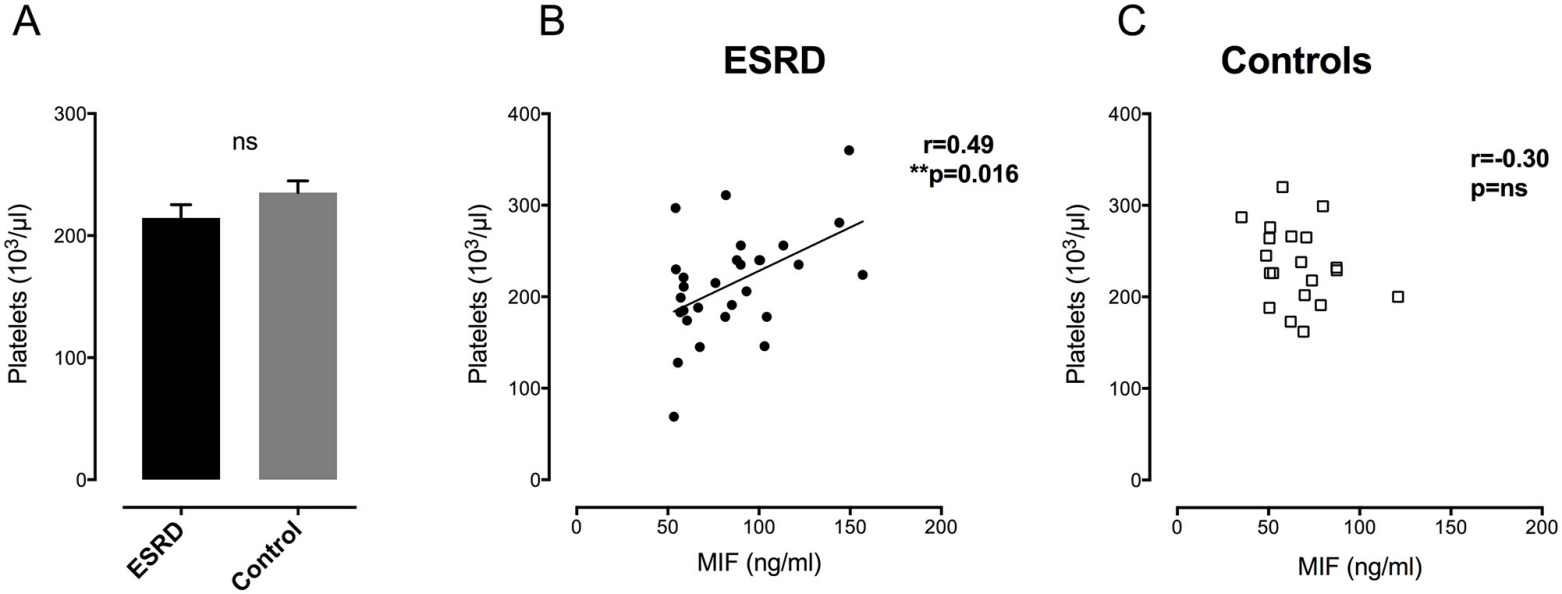
Hemodialysis removes large amounts of MIF from the circulating MIF plasma pool. Illustration of the experiment shows the estimated amount of circulating MIF levels in our patient cohort. Plasma levels were calculated being 5% of body weight (4.3L). Multiplication with the measured average MIF plasma level revealed an amount of 370 μg/patient. An average amount of 219±39 μg MIF was removed in a single HD session.

During hemodialysis, MIF levels distinctly decreased compared to pre-dialysis values in 27 out of 29 investigated individuals (MIF ESRD pre-HD 84.8±6 ng/ml to intra-HD 61.2±5 ng/ml, p<0.01, Fig 2A). Two individuals showed unaffected MIF levels without any significant change during or after HD. After termination of HD session, MIF plasma values returned to baseline levels (intra-HD 61.2±5 ng/ml to post-HD 79.8±5 ng/ml, p<0.01, Fig 2A). To further test whether decreased MIF levels are a consequence of hemoconcentration or volume shift after begin of the HD, we measured MIF in the ultrafiltrate (UF) fluid. Before HD, no MIF could be detected in the dialysis buffer as expected. In contrast, 30 min after initiation of HD, MIF could be detected in the UF fluid demonstrating that this protein is a plasma dialyzable component (Fig 2B). Late assessment of MIF in UF revealed equal concentration of MIF 30 minutes before the end of HD (Fig 2B). Except for two individuals out of 29, extend of MIF reduction during HD was tightly associated with baseline MIF plasma levels (r = 0.63; p<0.01 Fig 2C). To calculate the absolute amounts of removed MIF, we multiplied the measured MIF concentrations in the UF with the total amounts of dialysate that was applied during a single HD session. During a single HD session 73.7±1 L dialysate was used per patient and MIF content was determined in the ultrafiltrate (UF) (3.1±3 ng MIF/ml UF) (Fig 3).

We found that averagely 219±4 μg MIF was removed during a single HD-session per patient. To estimate the total amount of circulating MIF in a single patient, we calculated an average plasma volume of 5% body weight per patient (mean 4.3L). This results in an estimated amount of circulating MIF of 370 μg/patient (Fig 3).

### MIF plasma levels are associated with platelet count in ESRD patients

**Fig 4:**
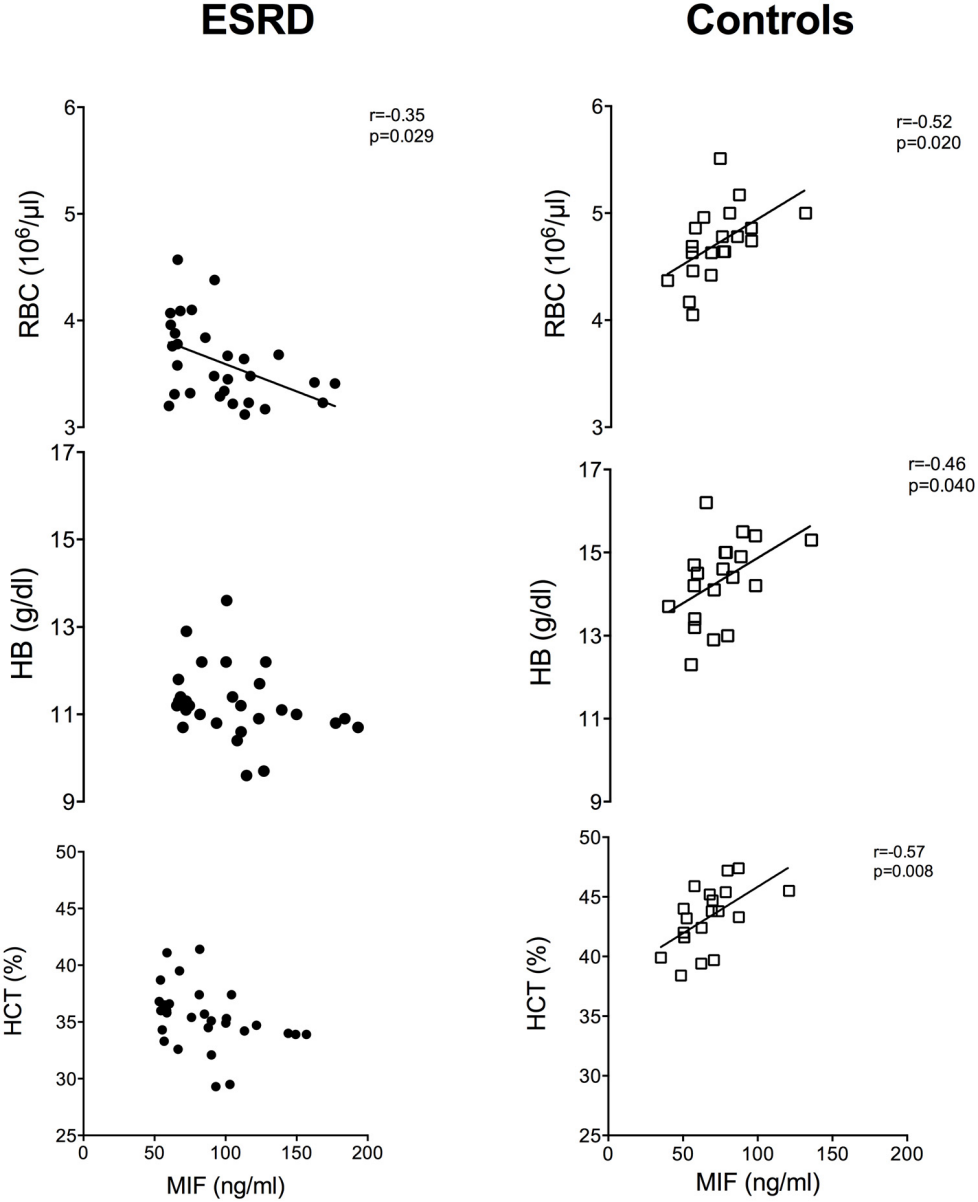
Platelets are associated with MIF plasma levels in ESRD patients but not in healthy controls. **A**) Platelets count does not differ between ESRD patients and healthy controls. **B**) Platelets count correlates with MIF plasma levels in ESRD patients (p<0.02). **C**) Healthy subjects show no correlation between platelets and circulating MIF levels (p = ns).

We found that large amounts of circulating MIF are removed during a single HD session and are reconstituted quickly after termination of HD (Figs 2 and 3). To test the hypothesis that MIF secretion from activated platelets might be a relevant finding *in vivo* that might account for this observation, MIF plasma levels and platelets count were determined in ESRD patients and in healthy, aged matched controls under baseline conditions. Platelet count showed no difference between ESRD patients and control individuals (Fig 4A).

In contrast, analysis of platelets count revealed that MIF plasma levels correlated with platelets in ESRD (r = 0.49, p<0.02, Fig 3B) whereas healthy controls exhibited no correlation of platelet count and MIF (Fig 4C). Thus, despite equal amounts of platelets, ESRD patients show a distinct correlation of MIF/platelets level compared to healthy controls.

### Parameters of acute inflammation show no relation to MIF plasma levels in ESRD patients

**Fig 5:**
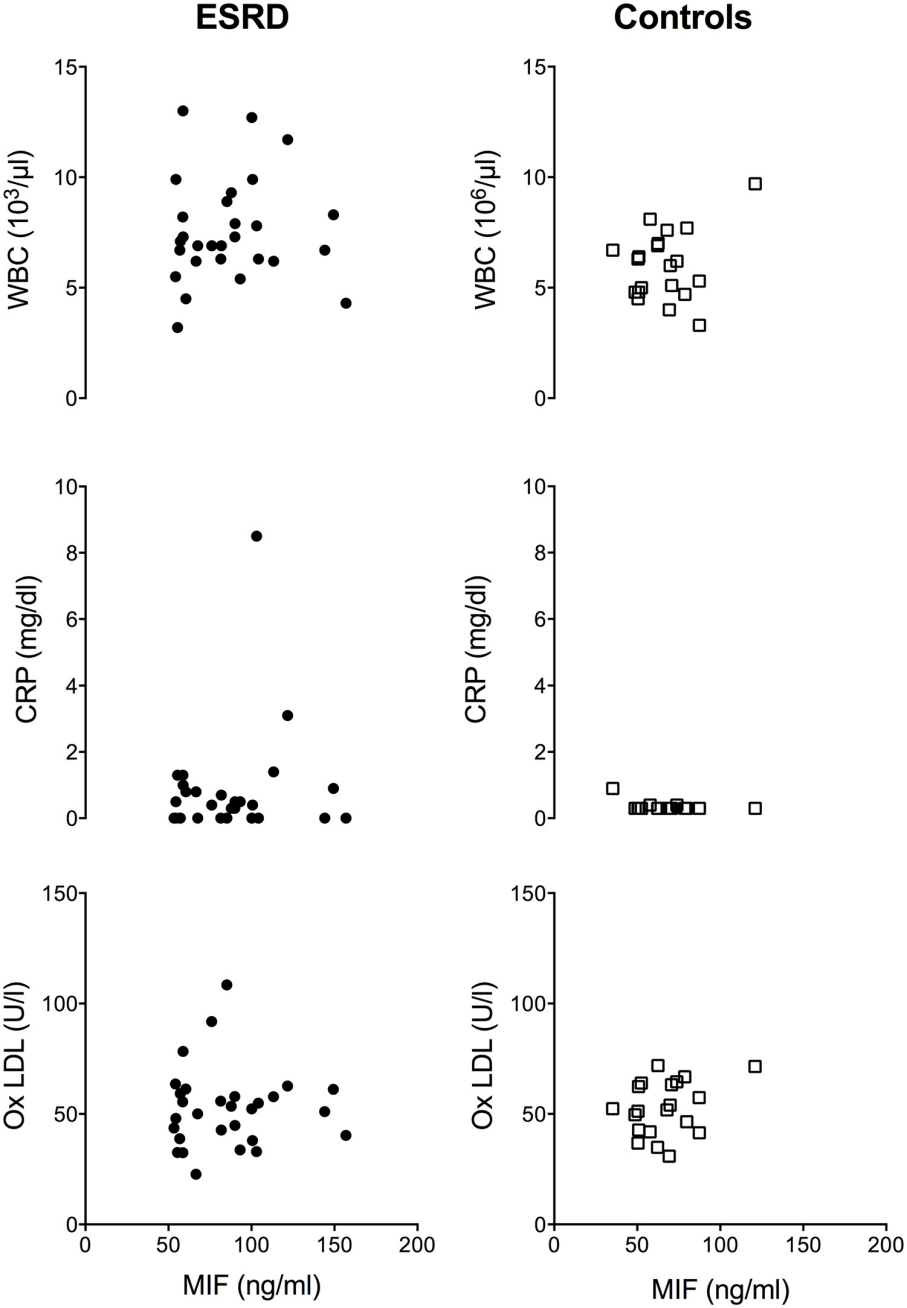
Increased MIF levels in ESRD show no association with inflammation. Inflammatory parameters (C reactive protein; CRP, white blood cells; WBC, oxidized LDL; oxLDL) were measured in ESRD after termination of HD and in healthy controls under resting conditions. Neither in ESRD nor in control MIF levels correlate with inflammatory parameters.

Since MIF is a mediator of inflammatory cell recruitment and MIF plasma levels are known to be elevated in inflammatory diseases like arthritis or sepsis, we analyzed parameters of inflammation in ESRD patients and controls. Analysis of C-reactive protein (CRP), white blood cells count (WBC) and oxLDL with MIF plasma levels revealed increased levels of these parameters in ESRD patients compared to healthy controls, confirming the chronic systemic inflammatory status of these patients (Fig 5).

Despite increased inflammatory markers, MIF plasma levels showed neither in ESRD nor in healthy controls correlation to CRP, WBC count, oxLDL (Fig 5). These data suggest that increased circulating MIF levels in ESRD disease patients are not a result of sustained inflammatory activity but might originate from other sources.

## Discussion

In this single-center prospective observational clinical study, we demonstrate that HD effectively removes MIF from the blood pool. Moreover we here show that ESRD patients exhibit an equilibrium of their MIF plasma levels that rapidly reconstitutes their basal MIF level after termination of HD. Tight correlation of platelets count with MIF plasma levels in our cohort suggests that MIF equilibrium might be a consequence of MIF secretion from chronically activated platelets in ESRD patients.

Our data underscore prior clinical findings that MIF plasma levels are elevated in chronic ill patients. These data are the first demonstrating that MIF plasma levels are decreased in a single HD session. In ESRD patients undergoing maintenance HD, the removal of circulating MIF could be advantageous in case of excess MIF formation. Concerning this matter, questions arise about the physiologic protein-bound fraction and possible HD-specific features, e.g. optimized filtration, absorption and removal by dialyzers. MIF is abundantly expressed in the vascular wall, in atherosclerotic lesions and has been shown being secreted by oxLDL- and hypoxia-stimulated endothelium and activated monocytes/macrophages.[12, 22–24] First evidence now demonstrates that MIF is expressed in human and murine platelets and is secreted from platelets upon activation.[25, 26] These data showed that platelets are a previously unrecognized source of MIF. It could be demonstrated that human and mouse platelets do not only contain significant amounts of MIF protein, but that they secrete MIF upon specific thrombogenic stimulation. So far, circulating MIF protein present in plasma after myocardial infarction or in atherogenic vessels has been considered being mainly secreted by endothelial cells, cardiomyocytes or circulating leukocytes. Now, one must consider platelet-derived MIF to contribute to the pool of circulating MIF in vivo. The physiological and pathophysiological significance of this MIF fraction is completely unknown. In our study, we observed that MIF levels rapidly recover after HD session. Since we did not measure any correlation between MIF and inflammatory parameters, one might rule out that replenishment of MIF was a consequence of an acute inflammatory response or release from inflammatory cells. Notably there was no correlation between MIF plasma levels and WBCs that contain large amounts of MIF. Thus, release of MIF from inflammatory cells can be ruled out in our setting. In contrast, tight correlation of MIF with platelet count supports latest findings demonstrating that platelets are previously unrecognized source of MIF. First reports on the role of platelets derived MIF demonstrated that this pro-inflammatory cytokine exerts an autocrine/paracrine effect on platelets and thereby regulates platelet survival and thrombotic properties.[25, 26] MIF was further found to be a major platelet-derived chemotactic recruitment factor with clot-modulating properties and therefore might be relevant in inflammatory diseases such as atherosclerosis.[27] These recent findings highlight MIF as a platelet derived chemokine, which is released upon platelet activation and that contributes to the circulating plasma pool of this cytokine. Stimulation of platelets during HD might be an explanation for the observed reconstitution of MIF plasma levels after termination of renal replacement therapy. It is an established fact that patients with chronic kidney disease exhibit increased platelet activation, and an attenuated response to dual antiplatelet therapy compared with patients without renal insufficiency.[9] Since we did not assess any parameters of platelet activation or analyzed platelet MIF content before and after HD, these data must be interpreted against this limitation of the study. To overcome this limitation in future studies, measurement of other factors that are almost exclusive platelet-origin and show similar size like the chemokine CXCL4, would support MIF as a platelet product. A promising experimental approach would be the ex vivo measurement of MIF release upon stimulation of platelets from ESRD patients and healthy controls. Nevertheless, despite limited sample size, single center and cross-sectional design our results are essential for the understanding of MIF-Kinetic in ESRD. The present data underscore the need to further enlighten the mechanisms and characterize the role of MIF and its prognostic impact in high-risk patients. Moreover, this is the first study demonstrating that application of HD can falsify MIF plasma measurement in patients. Since there are increasing amounts of clinical studies on the role of MIF in critically ill patients, frequently undergoing acute HD due to organ failure, it is of high importance to show that renal replacement therapy has an impact on MIF plasma level. [28–31]

In summary, we here show that MIF plasma levels are rapidly restored after removal from the blood pool in ESRD patients. MIF release from activated platelets might be a source of rapid MIF restoration in our cohort and should be further investigated in future studies. Renal replacement therapy can falsify MIF plasma level; this must be taken into consideration when evaluating the role of MIF in patients undergoing HD.

## Supporting Information

S1 Protocol (PDF)

S2 Protocol S1 and S2 files show the protocol of the underlying study investigating the influence of flavanols on vascular function in ESRD patients. (PDF)
