# Supplementary material for: Filtration of Macrophage Migration Inhibitory Factor (MIF) in Patients with End Stage Renal Disease Undergoing Hemodialysis
Source: PLoS One. 2015 Oct 20;10(10):e0140215. doi: 10.1371/journal.pone.0140215 (PMC4617461; doi:10.1371/journal.pone.0140215)
Supplement: S1 Protocol — (PDF) [file pone.0140215.s001.pdf]

Effekte einer flavanolreichen diätetischen Intervention auf die vaskuläre Funktion bei Patienten mit terminaler Niereninsuffizienz

Hauptprüfer

Universitätsprofessor Dr. med. Tienush Rassaf

Klinik für Kardiologie, Pneumologie und Angiologie

Universitätsklinikum Düsseldorf

Moorenstraße 5

40225 Düsseldorf

Prüfplan-Code: ESRD_2010

Version: K-XVII

Die Informationen in diesem Prüfplan sind streng vertraulich zu behandeln. Sie dienen nur zur Information des Prüfers, der Studienmitarbeiter, der Ethikkommission, und der Patienten. Dieser Prüfplan darf ohne Zustimmung des Sponsors oder des Hauptprüfers der klinischen Prüfung nicht an Dritte weitergegeben werden.

Unterschriften

**Prof. Dr. med. Tienush Rassaf**	______________________	__________
Hauptprüfer<br>Klinik für Kardiologie,<br>Pneumologie und Angiologie<br>Universitätsklinikum Düsseldorf	Unterschrift	Datum
**Prof. Dr. med. Malte Kelm**	______________________	__________
Direktor der Klinik für Kardiologie,<br>Pneumologie und Angiologie<br>Universitätsklinikum Düsseldorf	Unterschrift	Datum
**Prof. Dr. Christian Ohmann**	______________________	__________
Koordinierungszentrum für Klinische Studien<br>Universitätsklinikum Düsseldorf<br>Statistiker	Unterschrift	Datum
**Dr. med. Christos Rammos**	______________________	__________
Klinik für Kardiologie,<br>Pneumologie und Angiologie<br>Universitätsklinikum Düsseldorf<br>Prüfarzt	Unterschrift	Datum

I. Synopsis

Hauptprüfer:	Univ.-Prof. Dr. med. Tienush Rassaf
Titel der Studie:	Effekte einer flavanolreichen diätischen Intervention auf die vaskuläre Funktion bei Patienten mit terminaler Niereninsuffizienz
Bezeichnung der Prüfsubstanz:	Flavanolreiches Getränk und flavanolarmes Kontrollgetränk
Indikation	Verbesserung der Gefäßfunktion bei Patienten mit terminaler Niereninsuffizienz
Hauptprüfer und Prüfzentren	Prof. Dr. med. Tienush Rassaf<br>**Akutstudie:**<br>Klinik für Kardiologie, Pneumologie und Angiologie<br>Universitätsklinikum Düsseldorf<br>Moorenstr. 5<br>40225 Düsseldorf<br>und<br>**Langzeitstudie:**<br>Gemeinschaftspraxis Karlstraße<br>Bismarckstraße 101<br>40210 Düsseldorf
Zeitplan	**Akutstudie:**<br>Einschluss erster Patient (FPFV): 07/2011<br>Einschluss letzter Patient (Randomisierung) 08/2011<br>Prüfungsende des letzten Patienten (LPLV): 10/2011<br>Schließen der Datenbank: 11/2011<br>Ende der statistischen Auswertung: 11/2011<br>Integrierter Abschlussbericht:. 12/2011<br>**Langzeitstudie:**<br>Einschluss erster Patient (FPFV): 10/2011<br>Einschluss letzter Patient (Randomisierung) 06/2012<br>Prüfungsende des letzten Patienten (LPLV): 07/2012<br>Schließen der Datenbank: 08/2012

	Ende der statistischen Auswertung: 09/2012<br>Integrierter Abschlussbericht:. 10/2012
Art der Prüfung / Anzahl der Prüfzentren:	Monozentrisch / jeweils ein Prüfzentrum (Akut- und Langzeitstudie)
Endpunkte	**Akutstudie:**<br>• Wirksamkeit, Sicherheit und Dosis-Wirkungs-Beziehung einer flavanolreichen Diät bei Patienten mit terminaler Niereninsuffizienz an dialysefreien Tagen<br>**Langzeitstudie:**<br>Primäre Endpunkte:<br>• Verbesserung der Gefäßfunktion, endothelialen Regeneration sowie Reduktion von Gefäßschäden<br>Sekundäre Endpunkte:<br>• Wirksamkeit und Dosis-Wirkungs-Beziehung einer flavanolreichen Diät bei Patienten mit terminaler Niereninsuffizienz während der Hämodialyse<br>• Verbesserung von Lebensqualität, Durchblutung, Blutdrucksenkung und inflammatorischer Parameter
Methodik	**Akutstudie:**<br>Zweiarmige (flavanolreich und –arm), randomisierte, doppelblinde, Placebo kontrollierte Studie im Crossover-Design an 2 Untersuchungstagen<br>**Langzeitstudie:**<br>Zweiarmige (flavanolreich und –arm), randomisierte, doppelblinde, Placebo kontrollierte Studie im Parallel-Design
Patientenzahl	**Akutstudie:** 10 Patienten<br>**Langzeitstudie:** Insgesamt 52 Patienten (Je Arm 26)
Diagnose und Haupteinschlusskriterien:	Diagnose:<br>• Terminale chronische Niereninsuffizienz, Stadium V nach NKF KDOQI<br>Haupteinschlusskriterien:

	• >= 18 Jahre<br>• intermittierende chronische Hämodialyse<br>Hauptausschlusskriterien:<br>• Akutes Nierenversagen, Anurie, Hyperkaliämie, aktive maligne Erkrankung, Herzinsuffizienz (NYHA III-IV), akute Infektion (CRP>0,5 mg/dl), höhergradige Herzrhythmusstörung, Schwangerschaft,
Prüfsubstanz, Dosierung und Applikationsart:	Flavanolreiches Getränk, das zur Verfügung gestellte Pulver wird zweimal täglich aufgelöst in Wasser getrunken (820mg Flavanole).
Dauer der Therapie	**Akutstudie:** 2 Untersuchungstage, washout mit jeweils 1 Woche<br>**Langzeitstudie**: 30 ±5 Tage
Vergleichstherapie und Applikationsart:	Flavanolarmes Getränk, das Getränk wird nach Auflösung in Wasser getrunken (0mg Flavanole)
Statistiker	Prof. Dr. C. Ohmann<br>Koordinierungszentrum für Klinische Studien<br>Medizinische Fakultät, Heinrich-Heine-Universität<br>Moorenstr. 5<br>40225 Düsseldorf<br>Tel.: 0211-81-19700<br>Fax: 0211-81-19702<br>Email: ohmannch@uni-duesseldorf.de
Statistische Methoden:	Randomisierung:<br>Blockrandomisierung mit Blöcken variabler Länge, zentrale Randomisierung durch das KKS Düsseldorf<br>Auswertung der primäre Zielvariable:<br>• Vergleich der Prüfsubstanz mit Kontrolle:<br>t-Tests für unabhängige Stichproben<br>Auswertung der sekundäre Zielvariablen:<br>• Vergleich der Prüfsubstanz mit Kontrolle:<br>t-Test für unabhängige Stichproben (ggf. Mann-

	Whitney Test) bei quantitativen Variablen, Chi-Quadrat-Test bzw. exakter Test nach Fisher; bei kategorialen Daten.<br>• Weitere deskriptive und explorative Verfahren
GCP-Konformität:	Die vorliegende Prüfung wird gemäß der international anerkannten Good Clinical Practice-Leitlinie (ICH-GCP) einschließlich der Archivierung essentieller Dokumente durchgeführt
Finanzielle Förderung	Finanzierung durch Drittmittel der Klinik für Kardiologie, Pneumologie und Angiologie

II. Inhaltsverzeichnis

I. Synopsis 3

II. Inhaltsverzeichnis 7

III. Verzeichnis der Abbildungen 11

IV. Abkürzungsverzeichnis 12

1. Einleitung 13

2. Ziele der klinischen Prüfung 14

2.1. Rationale der klinischen Prüfung 14

2.2. Primäres Ziel 14

2.3. Sekundäre und weitere Ziele 15

3. Organisationsstruktur 16

3.1. Hauptprüfer 16

3.2. Statistik 16

3.3. Prüflabore und sonstige technische Einrichtungen 16

3.4. Zentrale Organisationseinheiten 16

3.5. Prüfer und Prüfzentren 16

3.6. Finanzierung 17

4. Studienplan 18

4.1. Allgemeines Studiendesign 18

4.2. Zeitplan 18

4.3. Diskussion des Studiendesigns 21

4.4. Auswahl der Studienpopulation 21

4.4.1. Einschlusskriterien 22

4.4.2. Ausschlusskriterien 22

4.4.3. Nachträglicher Ausschluss von Studienpatienten 23

4.5. Abbruch der gesamten Studie 23

4.6. Behandlungen 24

4.6.1. Angewendete Behandlungen 24

4.6.2. Beschreibung der Prüfsubstanz 24

4.6.2.1. Herstellung der Prüfsubstanz 24

4.6.2.2. Kennzeichnung der Prüfsubstanz 24

4.6.2.3. Lagerung der Prüfsubstanz 25

4.6.3. Einhaltung der Therapie /Ausgabe und Rücknahme der Prüfsubstanz 25

4.6.4. Methode zur Zuordnung der Patienten zu den Behandlungsgruppen 25

4.6.5. Auswahl der Dosierung der Prüfsubstanz 26

4.6.6. Festlegung von Dosierung und Zeitpunkt der Prüfsubstanzgabe für jeden Studienteilnehmer 26

4.6.7. Verblindung 26

4.6.7.1. Entblindung 26

4.7. Wirksamkeits- und Sicherheitsparameter 26

4.7.1. Messung der Wirksamkeits- und Sicherheitsparameter 26

4.7.1.1. Primärer Endpunkt, Sekundäre und weitere Endpunkt 26

4.7.1.2. Sicherheitsanalyse 26

4.7.1.3. Beschreibung der einzelnen Visiten 27

4.7.2. Wissenschaftliches Begleitprogramm 27

4.7.3. Pharmakokinetik 28

4.8. Sicherstellung der Datenqualität 28

4.8.1. Monitoring 28

4.8.2. Datenmanagement 29

4.8.3. Archivierung 29

5. Ethische und regulatorische Aspekte 31

5.1. Unabhängige Ethikkommissionen 31

5.1.1. Berücksichtigte gesetzliche Bestimmungen und Leitlinien 31

5.2. Registrierung 31

5.3. Aufklärung und Einverständnis der Studienteilnehmer 31

5.4. Patientenversicherung 32

5.5. Datenschutz 32

6. Statistische Methoden und Ermittlung der Fallzahl 33

6.1. Statistischer und analytischer Plan 33

6.1.1. Studienpopulationen 33

6.1.1.1. Intention-to-Treat-Population 33

6.1.1.2. Per-Protocol-Population 33

6.1.2. Beschreibung des Patientenkollektivs 33

6.1.3. Primäre Zielvariable 33

6.1.4. Sekundäre Zielvariablen 34

6.2. Ermittlung der Fallzahl 34

7. Unerwünschte Ereignisse 36

7.1. Mögliche Komplikationen und/oder Risiken 36

7.1.1. Mögliche Nebenwirkungen der Prüfsubstanz 36

7.1.2. Andere mögliche studienspezifische Komplikationen und/oder Risiken 36

7.1.3. Nutzen-Risiko-Abwägung 37

7.2. Kontrolle unerwünschter Ereignisse 37

7.3. Definitionen für unerwünschte Ereignisse 38

7.3.1. Unerwünschtes Ereignis 38

7.3.2. Schwerwiegendes unerwünschtes Ereignis 39

7.3.3. Zusammenhang des unerwünschten Ereignisses mit der Prüfsubstanz 39

7.3.4. Dokumentation von unerwünschten Ereignissen 40

7.4. Meldung von schwerwiegenden unerwünschten Ereignissen, 41

8. Verwendung der Daten und Publikation 42

8.1. Berichte 42

8.1.1. Abschlussbericht 42

8.2. Publikation 42

9. Literatur 43

10. Anhänge 44

A: Einwilligungserklärung 44

B: Einwilligungserklärung zum Datenschutz 46

C: Patienteninformation 48

D: Aufwandsentschädigung 55

E: Inhaltsstoffe der Prüfgetränke 56

III. Verzeichnis der Abbildungen

Abbildung 1: Ablaufdiagramm der klinischen Prüfung des Akutteiles mit Probanden an dialysefreien Tagen 19

Abbildung 2: Ablaufdiagramm der klinischen Prüfung der Langzeitstudie [Tag 1 und 30] mit Probanden während der Dialyse. 20

Abbildung 3: Ablaufdiagramm der klinischen Prüfung der Langzeitstudie, mit Probanden mit chronischer intermittierender Dialyse 21

IV. Abkürzungsverzeichnis

AB	Arteria brachialis
ACC	Arteria Carotis Comunis
AGEP	Advanced Glycation End Product
ADMA	Asymmetrisches Dimethylarginin
AE	Unerwünschtes Ereignis (Adverse Event)
CRP	C-reaktives Protein
CRF	Case Report Form, Erhebungsbögen
ED	Endotheliale Dysfunktion
eNOS	endotheliale NO-Synthase
EPC	Endothelial Progenitor Cells, endotheliale Vorläuferzellen
FMD	Flow-mediated Dilation, Fluss-abhängige Dilatation
GTN	Glyceroltrinitrat
HD	Hämodialyse
IL-6	Interleukin 6
iNOS	induzierbare NO-Synthase
IMT	Intima-Media-Thickness
LDL	Low density Lipoprotein
NO	Nitric Oxide, Stickstoffmonoxid
SAE	Schwerwiegendes unerwünschtes Ereignis (Serious Adverse Event)

1. Einleitung

Die terminale Nephropathie ist ein Problem von zunehmender gesundheitspolitischer Bedeutung mit einer jährlichen Inzidenz von 16.000 Neuerkrankungen in Deutschland. Patienten mit terminaler Niereninsuffizienz weisen ein stark erhöhtes kardiovaskuläres Risiko auf, bedingt durch fortschreitende strukturelle und funktionelle Veränderungen der arteriellen Leitungsgefäße sowie der Mikrozirkulation.

Die erhöhte Morbidität und Mortalität an Herz-Kreislauf-Erkrankungen bei Patienten mit einer chronischen Nierenkrankheit erklärt sich jedoch nur teilweise durch das vermehrte Vorkommen der traditionellen kardiovaskulären Risikofaktoren Diabetes Mellitus, Rauchen, arterielle Hypertonie und Hypercholersterinämie. Zusätzlich müssen auch Risikofaktoren, die mit der eingeschränkten Nierenfunktion oder deren Behandlung zusammenhängen wie eine endotheliale Dysfunktion, vaskuläre Kalzifizierung und chronische Inflammation in Betracht gezogen werden [1].

Die Ernährung stellt einen wichtigen modifizierbaren Lifestylefaktor dar, welcher die kardiovaskuläre Gesundheit wesentlich beeinflusst. Flavanole haben als pflanzliche Nahrungsbestandteile in diesem Zusammenhang besondere Aufmerksamkeit erreicht. Interventionsstudien bei Patienten mit kardiovaskulären Risikofaktoren wie Nikotinabusus und Diabetes Mellitus konnten zeigen, dass eine flavanolreiche Diät zu einer Verbesserung der Endothelfunktion und der Blutdruckeinstellung führt [2, 3]. Auf welchem Mechanismus diese vaskulären Effekte letztendlich beruhen und ob eine flavanolreiche Diät zu einer Verbesserung der Endothelfunktion bei Patienten mit terminaler Niereninsuffizienz führt, ist nicht bekannt.

Mit der vorliegenden Studie sollen folgende Ziele verfolgt werden: In der Akutstudie soll die Wirksamkeit, Sicherheit und die Dosis-Wirkungs-Beziehung eines Akut-Effektes nach einmaliger flavanolreicher diätischer Intervention bei niereninsuffizienten Patienten an hämodialysefreien Tagen erörtert werden.

In der Langzeitstudie soll schließlich der Effekt der Hämodialyse (HD) auf eine Flavanolreiche Diät sowie der Einfluss der diätischen Langzeiteinnahme eines flavanolreichen Getränkes auf die vaskuläre Funktion bei Patienten mit terminaler Niereninsuffizienz untersucht werden.

2. Ziele der klinischen Prüfung

2.1. Rationale der klinischen Prüfung

In vorangegangenen Interventionsstudien in Patienten mit kardiovaskulären Risikofaktoren, wie Diabetes mellitus oder Nikotinabusus, konnten gezeigt werden, dass eine flavanolreiche Diät zu einer Verbesserung der Gefäßfunktion führt [4, 5]. In dieser zweiteiligen Studie soll der Einfluss von Kakaoflavanolen bei Patienten mit terminaler Niereninsuffizienz untersucht werden.

Zunächst sollen in der **Akutstudie** Wirksamkeit, Sicherheit und die Dosis-Wirkungs-Beziehung nach Einnahme eines flavanolhaltigen Getränks bestimmt werden. Die Patienten erhalten, entsprechend dem Crossover-Design der Studie, abwechselnd an zwei unterschiedlichen Tagen ein flavanolhaltiges Getränk mit niedriger oder hoher Flavanoldosis. Vor der Einnahme und nach sechs Stunden wird das klinische Befinden untersucht, Blutproben zur Ermittlung des Flavanolmetabolismus werden jeweils zweistündlich entnommen und jeweils stündlich die vaskuläre Funktion mittels Ultraschalluntersuchungen ermittelt. Dies wird an dialysefreien Tagen untersucht. Zum Ende der Visite werden zusätzlich Blutgasanalysen zur Bestimmung der Elektrolytwerte und des Blut-ph Wertes bestimmt, um eine metabolische Derangierung der niereninsuffizienten Patienten nicht zu entgehen.

Anschließend soll in der **Langzeitstudie** die Verbesserung der Endothelfunktion bei Patienten mit terminaler Niereninsuffizienz, welche sich einer chronischen intermittierenden Hämodialyse unterziehen, untersucht werden. Die Patienten erhalten über 30 Tage eine flavanolreiche bzw. eine flavanolarme Diät. Zu Beginn und am Ende der diätischen Intervention wird sowohl das klinische Befinden als auch die Gefäßfunktion mittels Ultraschalluntersuchungen dargestellt und Blutproben zur Charakterisierung der endothelialen Funktion und Regeneration gewonnen. Weiterhin wird bei der klinischen Prüfung am ersten und letzten Tag genauso wie in der Akutstudie verfahren.

2.2. Primäres Ziel

Die Bestimmung der Wirksamkeit, Sicherheit und die Dosis-Wirkungs-Beziehung bei Patienten mit terminaler Niereninsuffizienz sind die primären Endpunkte in der Akutstudie.

Die Wirksamkeit einer flavanolreichen diätischen Intervention wird als Verbesserung der Gefäßfunktion definiert und mit der die Effektgröße FMD, als Surrogatparameter der endothelialen Funktion, bestimmt. Die Sicherheit der eingenommenen Prüfsubstanz wird duch laborchemische sowie klinische Untersuchungen sichergestellt. Im einzelnen sind dies Blutgasanalysen zur Bestimmung der Elektrolyte und des Blut pH-Wertes, hämodynamische Untersuchungen (Herzfrequenz und Blutdruck) und eine körperliche Untersuchung vor Abschluß der Visite. Die Dosis-Wirkungsbeziehung wird durch regelmäßige Messung des Blut-Flavanol-Gehaltes bestimmt und zur FMD korreliert.
In der Langzeitstudie ist der primäre Endpunkt die Verbesserung der vaskulären Funktion, gemessen an der FMD.

2.3. Sekundäre und weitere Ziele

In der Langzeitstudie sind die sekundären Endpunkte zum einen die Wirksamkeit und die Dosis-Wirkungs-Beziehung während der Hämodialyse sowie zum anderen Parameter, welche eine Verbesserung der Durchblutung, endothelialen Regeneration sowie Reduktion inflammatorischer Parameter widerspiegeln. Im Einzelnen sind dies Blutmarker und angiologische Funktions- und Gefäßstrukturuntersuchungen. Es wird erwartet, dass es zu einer Abnahme von Urämietoxinen wie AGEPs (advanced glycation end products), p-Cresol und β2-Mikroglobulin, von Inhibitoren der NO-Synthase wie Asymmetrisches Dimethylarginin (ADMA), von Entzündungsparametern (CRP, IL-6, oxidiertem LDL) und von Micropartikeln kommt. Eine Zunahme wird erwartet bezüglich der Expression der endothelialen und induzierbaren NO-Synthase, von NO-Metaboliten und EPCs. Eine verbesserte Durchblutung sollte sich sowohl in einer Steigerung der FMD (Makrozirkulation) als auch in einer verbesserten Mikrozirkulationsantwort zeigen. Strukturelle Verbesserungen sollten sich als Abnahme der Intima-Media Dicke, der arteriellen Steifigkeit und der Pulswellengeschwindigkeit sowie Zunahme der Compliance widerspiegeln. Weitere sekundäre Endpunkte umfassen eine verbesserte Blutdruckeinstellung und Verbesserung der Lebensqualität.

3. Organisationsstruktur

3.1. Hauptprüfer

Hauptprüfer:	Prof. Dr. med. T. Rassaf<br>Klinik für Kardiologie, Pneumologie und Angiologie<br>Universitätsklinikum Düsseldorf<br>Moorenstr. 5, 40225 Düsseldorf

3.2. Statistik

Statistiker:	Prof. Dr. C. Ohmann

3.3. Prüflabore und sonstige technische Einrichtungen

Labore:	Routine: Zentrallabor des Universitätsklinikums Düsseldorf<br>Bestimmung des Flavonolgehaltes:<br>Dr. K. Keen<br>University of California Davis<br>1 Shields Avenue,<br>Meyer Hall, Davis, CA, 95616 USA

3.4. Zentrale Organisationseinheiten

Monitoring:	Koordinierungszentrum für Klinische Studien<br>Universitätsklinikum Düsseldorf<br>Moorenstr. 5,<br>40225 Düsseldorf
Datenmanagement:	Koordinierungszentrum für Klinische Studien<br>Universitätsklinikum Düsseldorf<br>Moorenstr. 5<br>40225 Düsseldorf

3.5. Prüfer und Prüfzentren

Die Studie wird durch die Klinik für Kardiologie, Pneumologie und Angiologie monozentrisch durchgeführt in der

Klinik für Kardiologie, Pneumologie und Angiologie
Universitätsklinikum Düsseldorf
Moorenstr. 5
40225 Düsseldorf (Akutstudie)
und in der
Gemeinschaftspraxis Karlstraße
Bismarckstraße 101
40210 Düsseldorf (Langzeitstudie)

Hauptprüfer: Univ.-Prof. Dr. med. Tienush Rassaf
tienush.rassaf@med.uni-duesseldorf.de

Weitere Prüfer: Dr. med. Christos Rammos
christos.rammos@med.uni-duesseldorf.de

3.6. Finanzierung

Die Studie wird durch Drittmittel der Klinik für Kardiologie, Pneumologie und Angiologie finanziert.

Die Prüfungsteilnehmer erhalten nach jeweils abgeschlossener Studie eine Aufwandsentschädigung von 150 Euro (Akutstudie) bzw. 250 Euro (Langzeitstudie).

4. Studienplan

4.1. Allgemeines Studiendesign

Bei der Studie handelt es sich um eine Untersuchung im Sinne einer Verbindung von therapeutischer biomedizinischer Forschung am Menschen und Grundlagenforschung.

In der **Akutstudie** handelt es sich jeweils um eine zweiarmige (niedrig- und hochdosierte Flavanol-Diät), randomisierte, doppelblinde, kontrollierte Studie im Crossover-Design welche jeweils an 2 Tagen durchgeführt wird.

In der **Langzeitstudie** handelt es sich um eine zweiarmige (flavanolreich und –arm), randomisierte, plazebo-kontrollierte Interventionsstudie im Parallelgruppendesign.

4.2. Zeitplan

Zunächst soll die **Akutstudie** durchgeführt werden:

Einschluss erster Patient (FPFV):	07/2011
Einschluss letzter Patient (Randomisierung)	08/2011
Prüfungsende des letzten Patienten (LPLV):	10/2011
Schließen der Datenbank:	11/2011
Ende der statistischen Auswertung:	11/2011
Integrierter Abschlussbericht:.	12/2011

Nach Vollendung der Akutstudie wird die **Langzeitstudie** initiiert**:**

Einschluss erster Patient (FPFV):	10/2011
Einschluss letzter Patient (Randomisierung)	06/2012
Prüfungsende des letzten Patienten (LPLV):	07/2012
Schließen der Datenbank:	08/2012
Ende der statistischen Auswertung:	09/2012
Integrierter Abschlussbericht:.	10/2012
Prüfungsende	

Das Prüfungsende wird als letzte Visite des letzten Patienten angenommen und wird in der Akutstudie voraussichtlich bis 10/2011 und in der Langzeitstudie bis 07/2012 erreicht werden.

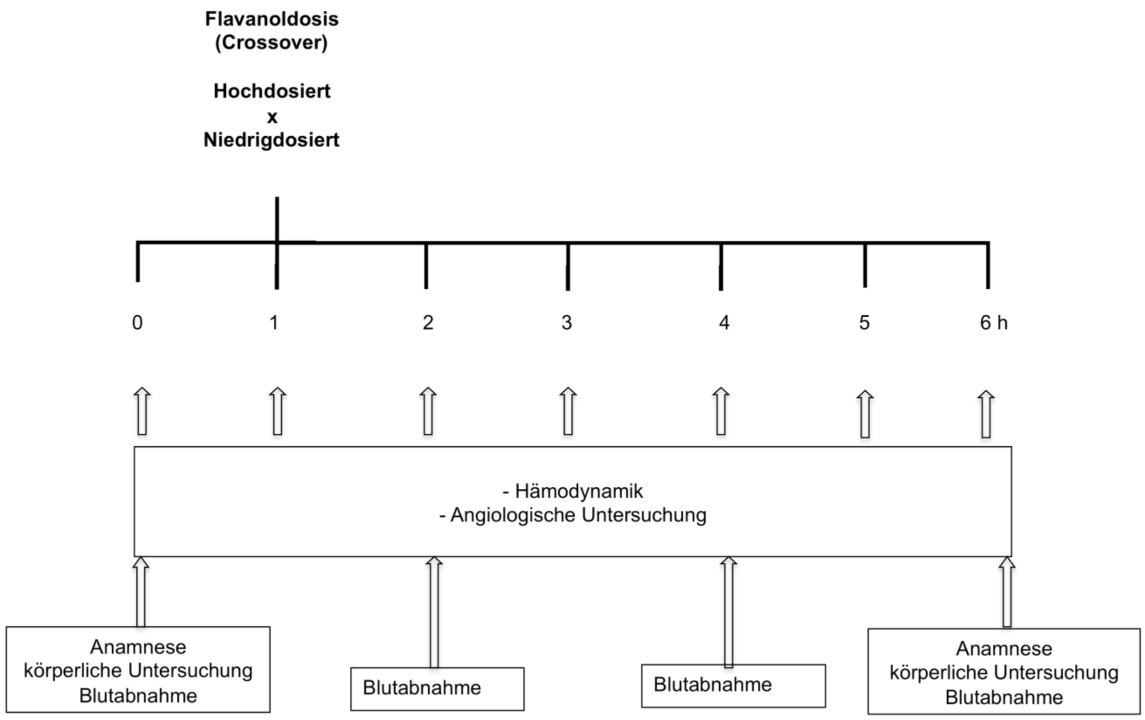

Abbildung 1: Ablaufdiagramm der klinischen Prüfung des Akutteiles mit Probanden an dialysefreien Tagen

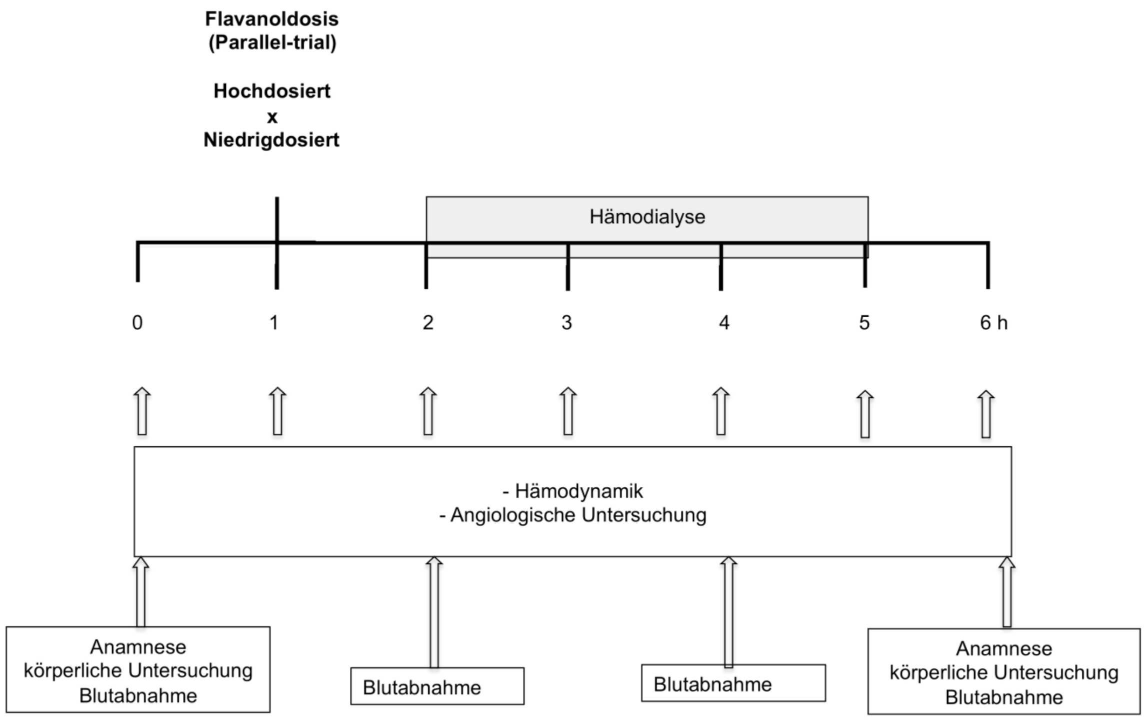

Abbildung 2: Ablaufdiagramm der klinischen Prüfung der Langzeitstudie [Tag 1 und 30] mit Probanden während der Dialyse.

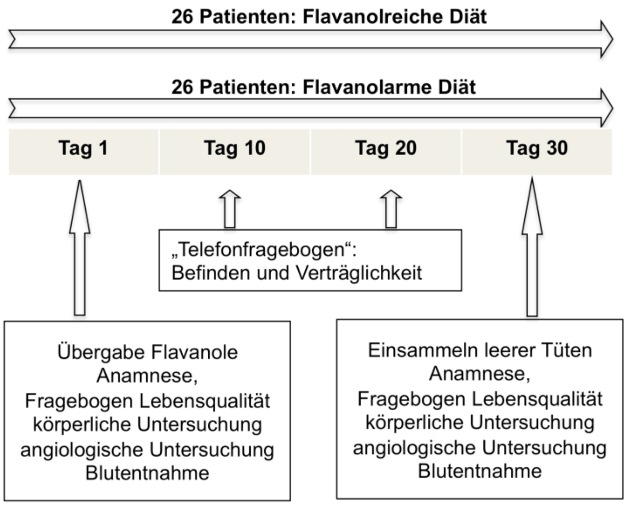

Abbildung 3: Ablaufdiagramm der klinischen Prüfung der Langzeitstudie, mit Probanden mit chronischer intermittierender Dialyse

4.3. Diskussion des Studiendesigns

Die Akut- sowie die Langzeitstudie wird zur Vermeidung von Bias doppelblind, randomisiert und plazebo-kontrolliert durchgeführt. Alle Patienten werden nach dem allgemeinen Standard ihres Krankheitsbildes therapiert.

4.4. Auswahl der Studienpopulation

Patienten mit terminaler Niereninsuffizienz weisen ein signifikant erhöhtes Risko für kardiovaskuäre Erkrankungen auf. Epidemiologische Daten zeigen einen konstanten Anstieg dieser Erkrankung auf. Die Mechanismen der ursächlichen endothelialen Dysfunktion in diesem Patientenkollektiv sind nicht abschließend geklärt [6]. Die Untersuchung erfolgt bei Patienten mit terminaler Niereninsuffizienz (Stadium V nach NKF/ KDOQI) gemessen an der glomerulären Filtrationsrate. Es werden nur Patienten mit chronischer intermittierender Hämodialyse (HD) als Nierenersatzverfahren eingeschlossen.

4.4.1. Einschlusskriterien

- Alter > 18 Jahre
- Geschäftsfähigkeit
- Terminale Niereninsuffizienz, Stadium V nach NKF/ KDOQI
- Chronische Intermittierende Hämodialyse als Nierenersatzverfahren

Studienteilnehmer, welche an der Akutstudie teilgenommen haben, können nach einer zeitlichen Latenz (washout mindestens 1 Woche) auch an der Langzeitstudie teilnehmen.

4.4.2. Ausschlusskriterien

- Akute Infektion
- Alter < 18 Jahre
- Anämie (Hb < 10 mg/dl)
- Persistierende Hyperkaliämie
- Anurie
- Maligne Erkrankung
- Herzinsuffizienz (NYHA III-IV)
- Höhergradige Herzrhythmusstörung
- maligne Grunderkrankung
- Teilnahme an anderen interventionellen Prüfungen, die mit der vorliegenden Prüfung interferieren könnten
- Beidseitige Arteria Brachialis Shunts
- Schwangerschaft oder Stillzeit
- Fehlende sichere Schwangerschaftsverhütungsmaßnahmen. Als sichere Schwangerschaftsverhütungsmaßnahmen gelten Verfahren mit einem Pearl-Index kleiner oder gleich 1 %:
    - Orale hormonelle Kontrazeption („Pille“),
    - Dermal hormonelle Kontrazeption,
    - Kontrazeptionspflaster,
    - Langzeit wirksame, injizierbare Kontrazeptiva,
    - Progestereon abgebendes Implantat (Impanon®),

- Progesteron i.m.
- Tubenligatur (weibliche Sterilisation),
- Hormon abgebendes Intrauterinpessar („Hormonspirale“),
- Doppelte Barrieremethoden

Als nicht zuverlässig gelten daher beispielsweise: Kondom plus Spermizid, einfache Barriere Methoden (Scheidenpessar, Kondom, weibliches Kondom, Kupferspirale, Rhythmusmethoden, Basaltemperaturmethode, Coitus interruptus).

4.4.3. Nachträglicher Ausschluss von Studienpatienten

Der Ausschluss von der Therapie geschieht bei Unverträglichkeit des Getränkes.
Ausgeschlossen von der Dokumentation werden Patienten, die sich als incompliant herausstellen. Eine Compliance wird als gegeben angesehen, wenn mindestens 93% des Prüfproduktes im vorgegeben Zeitfenster eingenommen wurden (an mindestens 28 von 30 Tagen der vorgegebenen Prüfprodukteinnahme).
Studienteilnehmer, die sich als incompliant herausstellen, werden in der Intention-to-treat-Analyse einbezogen.
Die Studienteilnahme erfolgt freiwillig. Jeder Teilnehmer kann sein Einverständnis zur Teilnahme zu jeder Zeit ohne Angabe von Gründen widerrufen, ohne dass ihm daraus Nachteile entstehen.

4.5. Abbruch der gesamten Studie

Die Studie wird insgesamt abgebrochen, wenn

- der Hauptprüfer aus Sicherheitsgründen einen Abbruch der Studie für notwendig erachtet,
- die Anwendung der Prüfsubstanz nicht länger vertretbar ist,
- die Studie sich als nicht durchführbar erweist.

Der Sponsor (Hauptprüfer) setzt sich zur Entscheidung zum Abbruch der Studie mit dem Biometriker ins Benehmen.

4.6. Behandlungen

4.6.1. Angewendete Behandlungen

Die Prüfungsteilnehmer trinken im Akutteil der Studie an zwei Untersuchungstagen jeweils niedrig- und hochdosierte flavanolhaltige Getränke. Im Langzeitteil der Studie soll an 30±5 Tagen jeweils zweimal täglich das flavanolarme oder -reiche Getränk eingenommen werden. Wie bereits vorab beschrieben, werden die Untersuchungen an den zwei Tagen der Akutstudie (Crossover-Design) und in beiden Behandlungsarmen der Langzeitstudie (Parallelgruppen-Design) jeweils identisch durchgeführt. Die Randomisierung der Studie erfolgt durch das Koordinationszentrum für klinische Studien unter der Leitung von Prof. Dr. rer. nat. C. Ohmann.

4.6.2. Beschreibung der Prüfsubstanz

4.6.2.1. Herstellung der Prüfsubstanz

Das verabreichte Getränk stellt ein natürliches und käuflich zu erwerbendes Nahrungsmittel dar (Inhaltsstoffe siehe Anhang E). In der gegenwärtigen Studie sollen Patienten über 30 Tage Getränke erhalten, welche reich (410mg) oder arm an Flavanolen (0mg) sind (kumulative Dosis/Tag 820mg Flavanole bzw. 0mg Flavanole). Die Testgetränke sind gematched bezüglich Makro-, Mikronährstoffen und Energiedichte. Die Zusammensetzung entspricht handelsüblichen Getränken. Ähnlich hohe Flavanolaufnahmen können in der täglichen Ernährung mit z.B. 1 l grünem Tee oder 2-6 Äpfeln erreicht werden. Der höhere Gehalt an Flavanolen wird durch besonders schonende Behandlung des Kakaoanteils erzielt (CocoaProTM), wobei eine schonendere Verarbeitung der Kakaobohnen zu einem höheren Flavanolgehalt führt. Die Komposition der Inhaltsstoffe ist in Anhang aufgeführt. Die Testgetränke werden von einer Stiftung (Mars Nutrition Research Council) gestellt und können käuflich erworben werden. Der Herstellungsprozess unterliegt den Standards welche für die Herstellung von Lebensmitteln gelten.

4.6.2.2. Kennzeichnung der Prüfsubstanz

In dieser Studie wird ein flavanolreiches Getränk mit einem flavanolarmen Kontrollgetränk gegenüber gestellt. Beide Getränke werden als Pulver in silberfarbenen Papiertüten verpackt

an die Patienten ausgegeben. Die Kennzeichnung erfolgt über einen aufgedruckten Zahlen oder Buchstabencode.

4.6.2.3. Lagerung der Prüfsubstanz

Die Lagerung der Prüf- und Kontrollgetränke erfolgt in den Räumlichkeiten der Forschungsgruppe unter der Leitung von Prof. Dr. med. M. Kelm.

4.6.3. Einhaltung der Therapie /Ausgabe und Rücknahme der Prüfsubstanz

In der Akutstudie erfolgt die Verabreichung der Kakaogetränke nach Randomisierungsplan des KKS durch den die Untersuchungen durchführenden Arzt am jeweiligen Untersuchungstag. Die Ausgabe der Testgetränke in der Langzeitstudie erfolgt am ersten Untersuchungstag ebenfalls nach Randomisierungsplan des KKS. Am Ende des jeweiligen Untersuchungstages (Akutstudie) und nach Abschluss der Langzeitstudie werden die leeren Papiertüten von den Patienten eingesammelt.

4.6.4. Methode zur Zuordnung der Patienten zu den Behandlungsgruppen

Nach ausführlicher Aufklärung und Einwilligung zur Studienteilnahme wird der Patient in einen der Behandlungsarme (Akutstudie Arm A/B: Prüfsubstanz a/b und Langzeitstudie Arm A: Prüfsubstanz, Arm B: Kontrollsubstanz) randomisiert. Dazu meldet der Prüfer Patienten montags bis freitags in der Zeit von 08:00 bis 16:00 Uhr per Fax unter der Faxnummer +49-211-8119705 an das KKS Düsseldorf. Nach Überprüfung des Fax (z.B. Ein-, Ausschußkriterien) erfolgt die Randomisierung im KKS Düsseldorf.

Grundlage dieser Zuordnung ist eine computergenerierte Block-Randomisierung unter Benutzung des Programmes „Rancode“. Der Randomisierungsplan wird durch das Koordinierungszentrum für Klinische Studien Düsseldorf erzeugt. Der Plan sieht eine 1:1 Ratio vor und permutiert in Blöcken von 4, 6 und 8 Patienten mit zufälliger Zuordnung.

4.6.5. Auswahl der Dosierung der Prüfsubstanz

In der gegenwärtigen Studie sollen Getränke (ein Getränk mit hoher (410 mg) oder niedriger Dosis an Flavanolen (0 mg) gegenüber gestellt werden. Die Testgetränke sind gematched bezüglich Makro und Mikronährstoffen und Energiedichte. Die Zusammensetzung entspricht handelsüblichen Getränken.

4.6.6. Festlegung von Dosierung und Zeitpunkt der Prüfsubstanzgabe für jeden Studienteilnehmer

Zur Auswahl und Zeitpunkt der Dosierung für jeden Studienteilnehmer wurde bereits im Abschnitt 4.6.1. ausführlich Stellung genommen.

4.6.7. Verblindung

Die Studie wird doppel-blind durchgeführt. Bis zur Entblindung bleibt die Identität der jeweiligen diätetischen Intervention geheim.

4.6.7.1. Entblindung

Ein Code mit der Entblindung der flavanolhaltigen Produkte wird bei Herrn Dr. med. M. Totzeck, Universitätsklinikum Düsseldorf, Klinik für Kardiologie, Pneumologie und Angiologie hinterlegt. Notfallkuverte werden von Herrn Dr. med. M. Totzeck, auf der Intensivstation und im Forschungslabor der Klinik hinterlegt. Zum Forschungslabor haben alle beteiligten Wissenschaftler jederzeit Zugang.

4.7. Wirksamkeits- und Sicherheitsparameter

4.7.1. Messung der Wirksamkeits- und Sicherheitsparameter

4.7.1.1. Primärer Endpunkt, Sekundäre und weitere Endpunkt

Zu primären, sekundären Endpunkten und weiteren Endpunkten wurde bereits unter 2.2 und 2.3 ausführlich Stellung genommen.

4.7.1.2. Sicherheitsanalyse

Im Rahmen der Studie werden unerwünschte Ereignisse erfasst und analysiert.

4.7.1.3. *Beschreibung der einzelnen Visiten*

Akutstudie und Langzeitstudie:

Die Visiten der Akutstudie und der Langzeitstudie gliedern sich identisch, lediglich wird nach der Einnahme des flavanolhaltigen Getränkes in der Langzeitstudie der Patient während der Hämodialyse untersucht (siehe Abbildung 2).

**Untersuchung**	**Zeitpunkt**	**Dauer**	**Untersucher**
Patientenaufklärungsbogen überprüfen	8.00 Uhr	10 Min	CR
Anamnese und körperliche Untersuchung	8.10 Uhr	20 Min	CR
Fragebogen zu Befindlichkeit und Ernährung	8.30 Uhr	20 Min	CR
Blutentnahme	8.00 Uhr<br>*je 2-Stunden*<br>-14.00 Uhr	10 Min	CR
FMD der AB, IMT der ACC, zeitgleich Blutdruckmessung beider Arme, Bestimmung der arteriellen Steifigkeit, Pulswellenanalyse, Nitroglyceringabe und Messung der endothelunabhängigen Vasodilatation der AB	9.00 Uhr<br>*stündlich*<br>-15.00 Uhr	30 Min	CR
Einnahme des flavanolhaltigen Getränkes	10:00 Uhr	10 Min	
Ggf Initiierung der HD (Langzeitstudie)	ab 10:00 Uhr		
Körperliche Untersuchung	15:30 Uhr	15 Min	CR
Ende	16:00 Uhr		

CR= Dr. med. C. Rammos

Dauer der Studie beim individuellen Patienten: Je Untersuchungstag ca. 8 Stunden.

4.7.2. Wissenschaftliches Begleitprogramm

Weitere Fragestellungen betreffen vor allem, über welche Mechanismen Flavanole positive Effekte auf die kardiovaskulare Gesundheit der Probanden vermitteln. Mechanistische Hinweise werden vor allem aus den Biomarkeranalysen sowie funktionellen und strukturellen angiologischen Untersuchungen gewonnen. Biomarker umfassen Marker des NO Stoffwechsels, Zytokine und zirkulierende Regenerationszellen. Angiologische

Untersuchungen sollen klären ob und wie sich die Makro und Mikrozirkulation verändert und ob es zu positiven Umbauvorgängen der Gefaßwände und verbesserter Elastizität der Arterien kommt. Weiterhin wird angenommen, dass es zu einer besseren Diabeteseinstellung und Blutdruckreduktion kommt.

4.7.3. Pharmakokinetik

Im Rahmen der Akutstudie werden vor der Einnahme der Flavanole sowie nach jeweils 2 Stunden NO-Metabolite und der Flavanolgehalt im periphervenösen Blut bestimmt (siehe Abbildung 1). Im Langzeitteil der Studie werden an Tag 1, also vor Beginn der täglichen Einnahme des flavanolhaltigen Getränks und am letzten Untersuchungstag entsprechend im 2 stündlichen Abstand Blutabnahmen durchgeführt zur Bestimmung der NO-Metabolite und des Flavanolgehaltes (siehe Abbildung 2).

4.8. Sicherstellung der Datenqualität

4.8.1. Monitoring

Zur Sicherstellung einer hohen Qualität der Studiendurchführung und der erhobenen Daten wird das Prüfzentrum regelmäßig durch Monitore besucht.

Bei einem zufällig ausgewählten Teil der Patienten wird ein möglichst vollständiger Abgleich der Daten in den Erhebungsbögen mit den Quelldaten vorgenommen. Bei jedem Patienten wird das Vorliegen des schriftlichen Einverständnisses als auch die Ein- und Ausschlusskriterien überprüft. Der genaue Umfang und die Art des Monitorings werden in einem gesonderten Monitoring-Manual beschrieben. In dem Manual werden alle studienspezifischen Belange erläutert und über Formblätter der Mindestumfang der Monitoringaktivitäten beschrieben.

Zwischen den Besuchen hält der zuständige Monitor regelmäßig telefonischen Kontakt mit dem Prüfzentrum. Diese Kontakte werden durch entsprechende Reports dokumentiert.

Alle Prüfer erklären sich damit einverstanden, dass der Monitor in regelmäßigen Abständen das Prüfzentrum besucht. Sinn und Zweck dieser Besuche sind insbesondere:

- die Evaluation des Studienfortgangs,
- die Kontrolle der Compliance mit dem Studienprotokoll,
- die Diskussion der Probleme einschließlich der AEs,

- die Überprüfung der CRFs auf Genauigkeit und Vollständigkeit,
- die Validierung der CRFs gegenüber den Originaldaten,
- die Überprüfung der Handhabung der Prüfsubstanz.

Über jeden Besuch wird ein Monitorbericht geführt, der den Fortschritt der Studie dokumentiert und über alle aufgetretenen Schwierigkeiten (z. B. Verweigerung der Einsichtnahme) unterrichtet.

Der Monitor hat das Recht, unter Berücksichtigung des Datenschutzgesetzes (der Monitor unterliegt der Schweigepflicht) die Prüfbögen mit den Originalunterlagen zu vergleichen (Krankenblätter, EKG, Laborausdrucke etc.). Die Prüfer ermöglichen dem Monitor für das prüfungsbezogene Monitoring direkten Zugang zu den Originaldaten-/ unterlagen.

4.8.2. Datenmanagement

Das Datenmanagement erfolgt mit Hilfe von Remote Data Entry (RDE). Die elektronische Case Report Form (eCRF) wird mit Hilfe eines modernen Clinical Data Managementsystems (CDMS) mit elektronischer Data Capture Funktionalität (EDC) im KKS Düsseldorf implementiert. Das System, eResearch Network™, erfüllt relevante internationale Standards und ermöglicht, die Durchführung des Datenmanagments innerhalb einer konsistenten, auditierbaren und integrierten elektronischen Umgebung (Query Managment, Dateneingabe, Datenvalidierung). Die Daten werden primär auf Papier-CRFs (pCRFs) gesammelt und dann vor Ort durch Studienpersonal eingegeben. Das Query Management wird elektronisch durchgeführt. Eingegeben werden nur pseudonymisierte Daten, die im KKS Düsseldorf gespeichert werden. Dabei werden geeignete Sicherheitstechniken eingesetzt. Nach Beendigung der Studie wir die Datenbank geschlossen und einschließlich des Audit-Trails archiviert. Für die statistische Analyse wird die geschlossene Datenbank in geeignete Formate exportiert.

4.8.3. Archivierung

Alle Dokumentationsbögen, Einverständniserklärungen sowie weitere wichtige Studienunterlagen werden gemäß gesetzlicher Grundlagen im Prüfzentrum archiviert. Die Patientenidentifikationsliste wird in allen Zentren getrennt von den Dokumentationsunterlagen aufbewahrt.

Die Archivierung der elektronischen Studiendatenbank im KKS Düsseldorf erfolgt nach den KKS eigenen SOPs auf der Grundlage gesetzlicher Regelungen.

5. Ethische und regulatorische Aspekte

5.1. Unabhängige Ethikkommissionen

Der vorliegende Prüfplan sowie ggf. nachfolgende Änderungen des Prüfplans wurden bzw. werden in Übereinstimmung mit der Deklaration von Helsinki in der Fassung vom Oktober 1996 (48th General Assembly of the World Medical Association, Somerset West, Republic of South Africa) verfasst.

Die Studie wird erst nach Vorliegen einer zustimmenden Bewertung der zuständigen Ethikkommission begonnen.

5.1.1. Berücksichtigte gesetzliche Bestimmungen und Leitlinien

Die Studie wird gemäß den ethischen Grundsätzen durchgeführt, wie sie in der Deklaration von Helsinki in der Fassung von 1996 (Sommerset) niedergelegt sind.

Sie wird in Übereinstimmung mit den in der Good Clinical Practice (ICH-GCP)-Leitlinie veröffentlichten Grundätzen und den zutreffenden gesetzlichen Bestimmungen durchgeführt. Diese Grundsätze betreffen unter anderem Ethikkommissions-Vorgänge, Patientenaufklärung und Einverständniserklärung, Befolgen des Protokolls, administrative Dokumente, Dokumentation der Prüfsubstanz, Datenerhebung, Patientenakte (Quelldokumente), Erfassung und Meldung unerwünschter Ereignisse (AE), Vorbereitung von Inspektionen und Audits sowie Aufbewahrung von Unterlagen. Alle Prüfer und weiteres unmittelbar mit der Prüfung befasstes Personal wurde darüber informiert, dass vom Sponsor der klinischen Prüfung autorisiertes Personal Studiendokumente und Patientenakten jederzeit einzusehen berechtigt sind.

5.2. Registrierung

Zu Beginn der Studie wird durch den Leiter der klinischen Prüfung die Registrierung der klinischen Prüfung bei einem öffentlich zugänglichen Studienregister veranlasst.

5.3. Aufklärung und Einverständnis der Studienteilnehmer

Ein Patient kann nur in die Studie aufgenommen werden, wenn er die Einwilligung hierzu erteilt hat, nachdem er durch einen Arzt mündlich und schriftlich über Wesen, Bedeutung und Tragweite der klinischen Prüfung in angemessener und verständlicher Weise aufgeklärt worden ist. Er muss mit der Einwilligung zugleich erklärt haben, dass er mit der im Rahmen

der klinischen Prüfung erfolgenden Aufzeichnungen von Daten und ihrer Überprüfung einverstanden ist. Der Patient wird über den potentiellen Nutzen und die Nebenwirkungen von Prüfsubstanz und Placebo sowie über die Notwendigkeit und Bedeutung einer placebokontrollierten Studie unterrichtet. Es muss ihm klar sein, dass er sein Einverständnis jederzeit und ohne Angabe von Gründen zurückziehen kann, ohne dass ihm hieraus Nachteile erwachsen.

Das Original der schriftlichen Einverständniserklärung wird im Studienordner des Prüfzentrums verwahrt. Dem Patienten wird eine Kopie der schriftlichen Patientenaufklärung sowie der Einverständniserklärung ausgehändigt. Zudem werden beide Dokumente in Kopie in der Patientenakte abgelegt.

Patienteninformationen zum Akuten und Chronischen Teil der Studie und die Einverständniserklärung sind als Anlage A-C beigefügt.

Patienteninformation und Einverständniserklärung werden der zuständigen Ethikkommission zur Begutachtung vorgelegt. Im Rahmen des Monitorings wird überprüft, ob die jeweils aktuelle Patienteninformation vor Beginn der Studie vom betroffenen Patienten unterzeichnet wurde.

5.4. Patientenversicherung

Eine Probandenversicherung wird für alle eingeschlossenen Patienten nach § 40 AMG mit einer Versicherung abgeschlossen werden. Für die Probanden der Akutstudie wird für die dialysefreien Untersuchungstage zusätzlich eine Wegeunfallversicherung abgeschlossen werden. Sitz, Police-Nr., Telefon- und Faxnummer der Versicherungsgesellschaft werden in die Patienteninformation aufgenommen (siehe dort).

5.5. Datenschutz

Die Bestimmungen des Datenschutzgesetzes werden beachtet. Es wird sichergestellt, dass alle Untersuchungsmaterialen und -daten entsprechend der Datenschutzbestimmungen vor wissenschaftlichen Verwertungen adäquat pseudonymisiert werden.

Die Prüfungsteilnehmer werden über die Weitergabe ihrer pseudonymisierten Daten im Rahmen der Dokumentations- und Mitteilungspflichten nach § 12 und § 13 GCP-V an die dort genannten Empfänger aufgeklärt. Personen, die der Weitergabe nicht zustimmen, werden nicht in die klinische Prüfung eingeschlossen werden.

6. Statistische Methoden und Ermittlung der Fallzahl

6.1. Statistischer und analytischer Plan

Für die statistische Auswertung wird ein separater Statistischer Analyseplan (SAP) erstellt. Dieser Plan muss vor Analysebeginn und vor der Entblindung vorliegen.

6.1.1. Studienpopulationen

6.1.1.1. Intention-to-Treat-Population

Dieser Datensatz enthält alle Patienten, die in die Studie aufgenommen und randomisiert wurden. Auch die Daten der Teilnehmer, die während der laufenden Studie ausscheiden – auf Wunsch der Teilnehmer, wegen Incompliance oder aus anderen Gründen- werden in die Auswertung miteinbezogen.

6.1.1.2. Per-Protocol-Population

Dieser Datensatz enthält alle Patienten, die über die gesamte Studiendauer dem Protokoll entsprechend behandelt wurden. Eine Compliance wird als gegeben angesehen, wenn mindestens 93% des Prüfproduktes im vorgegeben Zeitfenster eingenommen wurden (an mindestens 28 von 30 Tagen der vorgegebenen Prüfprodukteinnahme). Eine Abweichung vom Zeitplan im Rahmen der vorgegebenen Zeitfenster ist kein Grund für den Ausschluss aus dem PP-Datensatz.

Es wird für die Prüfung der Effektivität der Substanz das Signifikanzniveau bei a = 0,05 und die Power bei 80 % festgesetzt. Aufgrund der erwarteten Drop-out Rate von 5 % bis zum Ende von Phase III werden die Daten primär nach der Intention-to-treat-Methode analysiert.

6.1.2. Beschreibung des Patientenkollektivs

Die Beschreibung des Patientenkollektivs hinsichtlich demographischer Daten, Anamnese, klinischer Untersuchung sowie weiterer Baseline-Variablen erfolgt sowohl für die Gesamtgruppe als auch für die Substanzgruppen getrennt.

6.1.3. Primäre Zielvariable

Die primäre Zielvariable ist die „flow-mediated dilatation (FMD)“. Sie wird mit dem t-Test für unabhängige Stichproben geprüft.

6.1.4. Sekundäre Zielvariablen

Sekundäre Endpunkte, die den Vergleich der Substanzen betreffen, werden im Falle kontinuierlicher Variablen mit dem t-Test für unabhängige Stichproben (ggf. Mann-Whitney Test) und bei kategoriellen Daten mit dem Chi-Quadrat-Test bzw. mit dem exakten Test nach Fisher geprüft. Patienten mit fehlenden Daten werden von der Analyse ausgeschlossen. Darüberhinaus werden die Daten deskriptiv ausgewertet. Kontinuierliche Variablen werden durch Mittelwerte und Spannweiten beschrieben. Kategorielle Variablen werden durch die absoluten und relativen Häufigkeiten ihrer Kategorien beschrieben. Die unerwünschten Ereignisse werden nach klinisch sinnvollen Kategorien eingeteilt und für die Substanzgruppen dargestellt.

6.2. Ermittlung der Fallzahl

Die Fallzahlberechnung für die Langzeitstudie basiert auf dem primären Zielkriterium „flow-mediated dilatation (FMD)".

In einer früheren Studie bei Patienten mit koronarer Herzkrankheit [7] wurde gezeigt, dass die Differenz der FMD zwischen 30 Tagen nach Therapie und Baseline 1,3±2,0% (flavanolarm) und 3,8±2,67% (flavanolreich) betrug. Die geschätzte Effektgröße (Effektstärke) ist 0,897.

Bei einer anderen Studie bei Patienten mit Diabetes Mellitus [8] wurden separat für Kontrolle- und Treatmentgruppe zwischen 30 Tagen nach der Therapie und Baseline Differenzen der FMD von 0,1±1,15% und 1,0±1,15 ermittelt. Die geschätzte Effektgröße ist 0,782.

Daher wird in dieser Studie von einer durchschnittlichen Effektgröße von 0,840 ausgegangen. Unter Verwendung eines zwei Gruppen t-Tests für unabhängige Stichproben sowie einem Signifikanzniveau von 5%, zweiseitigem Test und einer Power von 80% ergibt sich eine Fallzahl von 24 Patienten pro Gruppe. (S. Tabelle).

Unter Berücksichtigung von ca. 5% Drop-outs resultiert insgesamt eine Fallzahl von 26 Patienten pro Gruppe.

Two group t-test of equal means (equal n's)

	1	2	3
**Test significance level, a**	0.050	0.050	0.050
**1 or 2 sided test?**	2	2	2
**Group 1 mean, m_1**			
**Group 2 mean, m_2**			
**Difference in means, m_1 - m_2**			
**Common standard deviation, s**			
**Effect size, d = $\|m_1 - m_2\|$ / s**	0.897	0.782	0.840
**Power (%)**	80	80	80
**n per group**	21	27	24

Die Fallzahlberechnung erfolgte mit dem Programm nQuery.

Für die Akutstudie wird die Fallzahl auf n=10 festgesetzt. Valide Zahlen für eine exakte Fallzahlschätzung liegen nicht vor.

7. Unerwünschte Ereignisse

7.1. Mögliche Komplikationen und/oder Risiken

7.1.1. Mögliche Nebenwirkungen der Prüfsubstanz

Das verabreichte Getränk stellt ein natürliches und käuflich zu erwerbendes Nahrungsmittel dar (Inhaltsstoffe siehe Anhang A). In der gegenwärtigen Studie sollen Patienten über 30 Tage ein Getränk erhalten, das reich (410 mg, kumulativ 820 mg/Tag) oder arm an Flavanolen (0 mg) ist. Die Testgetränke sind gematched bzg. Makro und Mikronährstoffen und Energiedichte. Die Zusammensetzung entspricht handelsüblichen Getränken. Ähnlich hohe Flavanolaufnahmen können in der täglichen Ernährung mit z.B. 1 l grünem Tee oder 2-6 Äpfeln erreicht werden. Der höhere Gehalt an Flavanolen wird durch besonders schonende Behandlung des Kakaoanteils erzielt (CocoaProTM). Wobei eine schonendere Verarbeitung der Kakaobohnen zu einem höheren Flavanolgehalt führt. Die Komposition der Inhaltsstoffen ist in Anhang E aufgeführt. Die Testgetränke werden von einer Stiftung (Mars Nutrition Research Council) gestellt und können käuflich erworben werden. Die unter der Nummer 2202 genehmigten Studien beinhalteten ähnliche Getränke. In den bisher durchgeführten Studien wurden die Testgetränke sehr gut toleriert und es kam bisher zu keinen unerwünschten Nebenwirkungen oder Komplikationen. Bei Patienten mit Diabetes mellitus und endothelialer Dysfunktion konnte nach Zufuhr von Kakaoflavanolen eine Verbesserung der vaskulären Funktion und keine negativen Effekte auf den Zuckerstoffwechsel nachgewiesen werden [8]. Auch kam es bisher im Verlauf keiner der anderen Studien zu kardiovaskulären Komplikationen [9, 10].

7.1.2. Andere mögliche studienspezifische Komplikationen und/oder Risiken

Blutentnahme: Im Rahmen der Untersuchungsreihe soll den Patienten an den Untersuchungstagen Blut aus einer Armvene entnommen werden. Zu den Risiken der Blutabnahme gehört das Entstehen von Hämatomen im Bereich der Einstichstelle. Es besteht das sehr geringe Risiko einer lokalen oder allgemeinen Infektion. In extrem seltenen Fällen kann es zu einer Verletzung eines Hautnervs, evtl. sogar mit chronischem Verlauf, kommen.

Angiologie: Die hochauflösende Dopplerultraschalluntersuchung ist ein nicht-invasives Verfahren um die sogenannte Fluss-mediierte Dilatation der Arterien (FMD) als Maß für die

endotheliale Funktion zu bestimmen. Hierzu wird der Durchmesser der Arteria brachialis vor und nach 5 minütiger Anlage einer Blutdruckmanschette am Oberarm mittels Ultraschall bestimmt. Die Anlage dieser Blutdruckmanschette verursacht keine Druckschäden und wird von den Probanden sehr gut toleriert. An Anlehnung an die gegenwärtigen Richtlinien zur Bestimmung der Endothelfunktion wird zur Abgrenzung einer glattmuskulären Dysfunktion mit vermindertem Ansprechen auf Stickstoffmonoxid im Anschluss an die FMD Untersuchung 0,4 mg Nitroglyzerin Zerbeißkapsel verabreicht [11]. Nach 4 Minuten wird der Durchmesser der Arteria brachialis erneut mittels Ultraschall bestimmt. Die potentiellen Nebenwirkungen dieses Medikaments sind transiente Kopfschmerzen und Blutdruckabfälle in hohen Dosierungen. Die Halbwertzeit des Medikamentes ist im Minutenbereich und Beinhochlagerung sowie Volumengabe sind die adäquate Therapie bis zum Abklingen der Wirkung. In der hier verwendeten Dosierung kam es bislang nie zu einem signifikanten Blutdruckabfall. Nichtsdestotrotz sind Transfusionsbestecke und Notfallmedikamente im Untersuchungsraum vorhanden. Hypotension und Unverträglichkeit stellen Kontraindikationen dar und sind deshalb Ausschlusskriterien. Die Untersuchungen werden von Dr. med. Rammos durchgeführt und vor dem Aufstehen von der Untersuchungsliege wird der Blutdruck gemessen. Mit Votum der hiesigen Ethikkommission konnte dieses Verfahren bereits mehrfach eingesetzt werden (Antrag 2202).

7.1.3. Nutzen-Risiko-Abwägung

Diese klinische Studie untersucht den Effekt von Flavanol in einem Ernährungsmittel auf die kardiovaskuläre Gesundheit von Patienten mit terminaler Niereninsuffizienz. Die Hypothese, die Verbesserung der Gefäßfunktion, hat einen direkten Effekt auf die Gesundheit und die Lebensqualität der Patienten. Aufgrund dessen ist von einem hohen Nutzen dieser Untersuchung auszugehen. Risiken der Behandlung sind in keiner der vorangegangenen Untersuchungen aufgetreten und werden auch in dieser Studie nicht erwartet. Dementsprechend ist das Risiko der Studie als gering einzustufen.

7.2. Kontrolle unerwünschter Ereignisse

Der Hauptprüfer trägt dafür Sorge, dass alle Personen, die an der Behandlung der Studienpatienten beteiligt sind, adäquat über die Verantwortlichkeiten bei Auftreten unerwünschter Ereignisse informiert sind. Bei jeder Visite werden die Patienten befragt, ob

unerwünschte oder schwerwiegende unerwünschte Ereignisse aufgetreten sind. Unerwünschte Ereignisse werden sowohl in der Patientenakte als auch in den Erhebungsbögen dokumentiert. Im Rahmen der Visiten wird die Patientenakte nach stattgehabten unerwünschten Ereignissen geprüft.

Bei Auftreten eines unerwünschten Ereignisses muss der betreffende Patient, unabhängig vom Kausalzusammenhang zwischen unerwünschten Ereignis und Prüfpräparat, in jedem Fall solange beobachtet werden, bis die Symptome abgeklungen sind, pathologische Laborwerte auf die Ausgangswerte zurückgegangen sind, sich eine plausible Erklärung für das unerwünschte Ereignis ergeben hat oder bis zum Tode des Patienten.

7.3. Definitionen für unerwünschte Ereignisse

7.3.1. Unerwünschtes Ereignis

Unerwünschtes Ereignis ist jedes nachteilige medizinische Vorkommnis, das einer betroffenen Person widerfährt, der ein Prüfpräparat verabreicht wurde, und das nicht notwendigerweise in ursächlichem Zusammenhang mit dieser Behandlung steht.

Begleiterkrankungen

Auch die Verschlechterung einer vorbestehenden Erkrankung ist in diesem Zusammenhang als unerwünschtes Ereignis anzusehen. Als unerwünschtes Ereignis gilt jedoch nicht eine Maßnahme zur Behandlung einer vorbestehenden Erkrankung, die bereits vor Einschluss in der Studie geplant war.

Schwangerschaft

Das Eintreten einer Schwangerschaft gilt im Rahmen dieser Studie aus Gründen der Sicherheit als unerwünschtes Ereignis.

Intensität

Der Prüfer wird im Verlauf der Prüfung feststellen, ob unerwünschte Ereignisse aufgetreten sind und wird ihre Intensität wie folgt einstufen:

- Leicht: Klinisches Symptom oder Zeichen, das gut toleriert wird.
- Mittel: Klinisches Symptom oder Zeichen, das ausreichend ist, die normale Aktivität zu beeinträchtigen.
- Schwer: Klinisches Symptom oder Zeichen, das zu einer starken Beeinträchtigung oder zur Arbeitsunfähigkeit oder der Unfähigkeit, alltägliche Verrichtungen durchzuführen führt.

7.3.2. Schwerwiegendes unerwünschtes Ereignis

Ein schwerwiegendes unerwünschtes Ereignis (Serious Adverse Event, SAE) ist jedes Ereignis, das

1. tödlich oder lebensbedrohend ist,
2. eine stationäre Behandlung oder deren Verlängerung erforderlich macht,
3. zu bleibender oder schwerwiegender Behinderung oder Invalidität führt,
4. eine kongenitale Anomalie oder einen Geburtsfehler zur Folge hat oder
5. jedes andere Ereignis, das ein vergleichbares Kriterium erfüllt (nach Maßgabe des beurteilenden Prüfers).

Als lebensbedrohlich im obigen Zusammenhang werden Ereignisse betrachtet, bei denen die Gefahr zu sterben zum Zeitpunkt des Ereignisses bestand.

Als Krankenhausaufnahme wird jeder stationäre Aufenthalt eines Patienten angesehen, der mindestens eine Nacht (0 – 6 Uhr) umfasst hat. Bereits vor der ersten Gabe des Prüfpräparates geplante Krankenhausaufnahmen gelten nicht als schwerwiegende unerwünschte Ereignisse.

Wenn ein unerwünschtes Ereignis als schwerwiegend eingeschätzt wird, wird das Ereignis zusätzlich zur AE-Dokumentation auf einem gesonderten SAE-Bogen dokumentiert.

7.3.3. Zusammenhang des unerwünschten Ereignisses mit der Prüfsubstanz

Jedes unerwünschte Ereignis wird vom Prüfer beurteilt, ob ein Zusammenhang mit der Prüfsubstanz vermutet werden kann oder nicht. Dabei müssen die Art und das Muster der Reaktion, der zeitliche Zusammenhang zur Verabreichung der Studienmedikation, der klinische Status des Patienten, die Begleitmedikation und andere relevante klinische Parameter in Betracht gezogen werden.

Für die Kausalitätsbeurteilung des unerwünschten Ereignisses mit dem Prüfprodukt gelten folgende Begriffsbestimmungen:

- Sicher: Ein Ereignis, das einem nachvollziehbaren zeitlichen Ablauf nach der Anwendung des Prüfproduktes folgt oder bei der die Prüfproduktkonzentration in Körpergewebe oder -flüssigkeit gemessen wurde, einem bekannten oder erwarteten Antwortmuster auf das verdächtigte Prüfprodukt folgt und nach

Absetzen oder Dosisreduktion verschwindet und bei erneuter Exposition wieder auftritt.

- Wahrscheinlich: Ein Ereignis, das einem nachvollziehbaren zeitlichen Ablauf nach der Anwendung des Prüfproduktes folgt, einem bekannten oder erwarteten Antwortmuster auf das verdächtigte Prüfprodukt folgt und nach Absetzen oder Dosisreduktion verschwindet und nicht durch die bekannten Merkmale des klinischen Zustandes des Probanden/Patienten erklärt werden kann.
- Möglich: Ein Ereignis, das einem nachvollziehbaren zeitlichen Ablauf nach der Anwendung des Prüfproduktes folgt, einem bekannten oder erwarteten Antwortmuster auf das verdächtigte Prüfprodukt folgt, die aber leicht auch durch eine Reihe anderer Faktoren hervorgerufen worden sein könnte.
- Kein Zusammenhang: Ein Ereignis, bei dem ausreichend Informationen vorliegen für die Annahme, dass kein Zusammenhang mit dem Prüfprodukt besteht.
- Nicht beurteilt: Ein Ereignis, das als unerwünschtes Ereignis gemeldet wurde, bei dem eine Beurteilung des Zusammenhangs zum Zeitpunkt der Meldung nicht erfolgt ist, weil weitere Daten notwendig sind oder zurzeit erhoben werden.
- Nicht beurteilbar: Eine Einschätzung des Zusammenhangs ist nicht möglich.

7.3.4. Dokumentation von unerwünschten Ereignissen

Alle unerwünschten Ereignisse werden im CRF dokumentiert. Unerwartete unerwünschte Ereignisse werden im CRF einschließlich folgender Parameter erfasst:

- Datum und Zeit des Beginns und Endes,
- Schwere,
- Zusammenhang mit dem Prüfpräparat,
- Schwerwiegend oder nicht-schwerwiegend,
- Erwartet oder unerwartet,
- Unterbrechung oder Absetzen der Prüfpräparatgabe.

Bei Auftreten eines unerwünschten Ereignisses muss der betreffende Patient, unabhängig vom Kausalzusammenhang zwischen AE und Prüfpräparat, in jedem Fall solange beobachtet werden, bis die Symptome abgeklungen sind, pathologische Laborwerte auf die Ausgangswerte zurückgegangen sind, sich eine plausible Erklärung für das unerwünschte

Ereignis ergeben hat, bis zum Tode des Patienten oder die klinische Prüfung bei dem betroffenen Patienten beendet ist.
Erkrankungen, die vor Verabreichung des Prüfpräparates bestanden, werden nicht als AE sondern als Begleiterkrankung dokumentiert. Jede neue Erkrankung oder jede Erkrankung mit zunehmender Schwere wird im Verlauf der Studie als AE dokumentiert.

7.4. Meldung von schwerwiegenden unerwünschten Ereignissen,

Jedes schwerwiegende unerwünschte Ereignis im Verlauf der Studie muss im entsprechenden CRF-Teil dokumentiert werden.
Die Prüfer informieren umgehend den Hauptprüfer nach Bekanntwerden eines SAE mit dem dafür vorgesehenen Dokumentationsbogen unter der Telefonnummer 02118118800 und der Email-Adresse tienush.rassaf@med.uni-duesseldorf.de.
Die Prüfer informieren ebenso umgehend über eine im Verlauf der klinischen Prüfung aufgetretenen Schwangerschaft und ihrem Ausgang.

8. Verwendung der Daten und Publikation

8.1. Berichte

8.1.1. Abschlussbericht

Der Hauptprüfer informiert die Ethikkommission über die Beendigung der klinischen Prüfung.

8.2. Publikation

Es ist vorgesehen, die Ergebnisse der klinischen Prüfung zu gegebener Zeit und nach gegenseitiger Abstimmung mit dem Leiter der klinischen Prüfung in einer wissenschaftlichen Fachzeitschrift und/oder bei deutschen und internationalen Kongressen vorzustellen. Grundsätzlich ist einer Gesamtpublikation der klinischen Prüfung Vorzug zu geben. Die „Uniform requirements for manuscripts submitted to biomedical journals. International Committee of Medical Journal Editors" [JAMA 1997;277:927-34] werden berücksichtigt.
Für alle Veröffentlichungen gilt, dass der Datenschutz sowohl für alle Patientendaten als auch für die Daten der teilnehmenden Ärzte gewahrt bleibt.

9. Literatur

1. Go, A.S., et al., *Chronic kidney disease and the risks of death, cardiovascular events, and hospitalization.* N Engl J Med, 2004. **351**(13): p. 1296-305.

2. Heiss, C., C.L. Keen, and M. Kelm, *Flavanols and cardiovascular disease prevention.* Eur Heart J, 2010. **31**(21): p. 2583-92.

3. Arts, I.C. and P.C. Hollman, *Polyphenols and disease risk in epidemiologic studies.* Am J Clin Nutr, 2005. **81**(1 Suppl): p. 317S-325S.

4. Heiss, C., et al., *Vascular effects of cocoa rich in flavan-3-ols.* JAMA, 2003. **290**(8): p. 1030-1.

5. Heiss, C., et al., *Endothelial function, nitric oxide, and cocoa flavanols.* J Cardiovasc Pharmacol, 2006. **47 Suppl 2**: p. S128-35; discussion S172-6.

6. Kao, M.P., et al., *Oxidative stress in renal dysfunction: mechanisms, clinical sequelae and therapeutic options.* J Hum Hypertens, 2010. **24**(1): p. 1-8.

7. Heiss, C., et al., *Improvement of endothelial function with dietary flavanols is associated with mobilization of circulating angiogenic cells in patients with coronary artery disease.* J Am Coll Cardiol, 2010. **56**(3): p. 218-24.

8. Balzer, J., et al., *Sustained benefits in vascular function through flavanol-containing cocoa in medicated diabetic patients a double-masked, randomized, controlled trial.* J Am Coll Cardiol, 2008. **51**(22): p. 2141-9.

9. Heiss, C., et al., *Acute consumption of flavanol-rich cocoa and the reversal of endothelial dysfunction in smokers.* J Am Coll Cardiol, 2005. **46**(7): p. 1276-83.

10. Schroeter, H., et al., *(-)-Epicatechin mediates beneficial effects of flavanol-rich cocoa on vascular function in humans.* Proc Natl Acad Sci U S A, 2006. **103**(4): p. 1024-9.

11. Corretti, M.C., et al., *Guidelines for the ultrasound assessment of endothelial-dependent flow-mediated vasodilation of the brachial artery: a report of the International Brachial Artery Reactivity Task Force.* J Am Coll Cardiol, 2002. **39**(2): p. 257-65.

10. Anhänge

A: Einwilligungserklärung

Universitätsklinikum Düsseldorf
Klinik für Kardiologie, Pneumologie und Angiologie
Direktor: Univ.- Prof. Dr. M. Kelm

Einwilligungs-/ Einverständniserklärung

Für die Teilnahme an der Studie:

“Effekte einer flavanolreichen diätischen Intervention auf die vaskuläre Funktion bei Patienten mit terminaler Niereninsuffizienz”

Ich

Name: .... Vorname: ....

Geburtsdatum: .... Telefon: ....

Strasse: .... PLZ: ....

erkläre mich hiermit bereit, an der Studie „Effekte einer flavanolreichen diätischen Intervention auf die vaskuläre Funktion bei Patienten mit terminaler Niereninsuffizienz” des Universitätsklinikums Düsseldorf, teilzunehmen. In dieser Studie soll untersucht werden, welchen Einfluß Flavanole, die sich natürlicherweise in bestimmten Lebensmitteln, wie Obst, Gemüse, Tee, Rotwein und auch in Kakaobohnen befinden, auf die Gefäßfunktion haben.

Ich wurde über die Einzelheiten (Zweck, Anforderungen und Risiken) der Studie im Rahmen der beiliegenden Patienteninformation unterrichtet und hatte ausreichend Möglichkeiten, Fragen über den Hintergrund der Studie und über den voraussichtlichen Nutzen zu stellen.

Ich wurde darüber unterrichtet, dass in der Studie Fragen über mich, meine Gesundheit und Lebensgewohnheiten gestellt, sowie Blutuntersuchungen, nichtinvasive Untersuchungen der Gefäße durchgeführt werden. Über etwaige, selten auftretende Nebenwirkungen durch Blutabnahmen, Medikamente, oder einen nicht zu erwartenden Anstieg des Blutkaliumwertes durch das Prüfpräparat bin ich aufgeklärt worden.

Ich erkläre mich hiermit einverstanden, dass meine Krankengeschichte, meine persönlichen Angaben und die Ergebnisse der Untersuchungen, die während der Studie erhoben werden, von autorisierten Personen eingesehen werden dürfen -vorausgesetzt, sie sind kodiert, das heißt pseudonymisiert, so dass eine Zuordnung der Ergebnisse zu meinem Namen und meiner Person durch Außenstehende nicht möglich ist.

Mir ist bekannt, dass ich die Teilnahem an der Studie jederzeit, ohne Angabe von Gründen und ohne Nachteile für meine medizinische Behandlung, widerrufen kann.

Datum: .... Unterschrift (Patient): ....

Datum: .... Unterschrift (Prüfarzt): ....

B: Einwilligungserklärung zum Datenschutz

Universitätsklinikum Düsseldorf
Klinik für Kardiologie, Pneumologie und Angiologie
Direktor: Univ.- Prof. Dr. M. Kelm

"Effekte einer flavanolreichen diätischen Intervention auf die vaskuläre Funktion bei Patienten mit terminaler Niereninsuffizienz"

Mir ist bekannt, dass bei dieser Studie personenbezogene Daten, insbesondere medizinische Befunde, über mich erhoben, gespeichert und ausgewertet werden sollen. Die Verwendung der Angaben über meine Gesundheit erfolgt nach gesetzlichen Bestimmungen und setzt vor der Teilnahem an der Studie folgende freiwillig abgegebene Einwilligungserklärung voraus, d.h. ohne die nachfolgende Einwilligung kann nicht an der Studie teilgenommen werden.

Einwilligungserklärung zum Datenschutz

1) Ich erkläre mich hiermit einverstanden, dass im Rahmen dieser Studie erhobene Daten, insbesondere Angaben über meine Gesundheit, erhoben, in Papierform oder auf elektronischen Datenträgern in der Klinik für Kardiologie, Pneumologie und Angiologie aufgezeichnet und gespeichert werden. Soweit erforderlich, dürfen die erhobenen Daten pseudonymisiert (verschlüsselt) an andere Institute für Analysen weitergegeben werden.

2) Ich bin darüber aufgeklärt worden, dass ich meine Einwilligung zur Aufzeichnung, Speicherung und Verwendung meiner Daten jederzeit widerrufen kann. Bei einem Widerruf werden meine Daten unverzüglich gelöscht.

3) Ich erkläre mich hiermit damit einverstanden, dass meine Daten nach Beendigung oder Abbruch der Studie 2 Jahre aufbewahrt werden. Danach werden meine personenbezogenen Daten gelöscht, soweit dem nicht gesetzliche, satzungsgemäße oder vertragliche Aufbewahrungsfristen entgegenstehen.

Name: .... Vorname: ....

Datum: .... Unterschrift (Patient): ....

C: Patienteninformation

Universitätsklinikum Düsseldorf
Klinik für Kardiologie, Pneumologie und Angiologie
Direktor: Univ.- Prof. Dr. M. Kelm

Patienteninformation

Für die Teilnahme an der Studie:

"Effekte einer flavanolreichen diätischen Intervention auf die vaskuläre Funktion bei Patienten mit terminaler Niereninsuffizienz"

Sehr geehrte Teilnehmerin, sehr geehrter Teilnehmer,

Wir laden Sie ein, an der oben genannten Studie teilzunehmen. Die Aufklärung darüber erfolgt in einem ausführlichen ärztlichen Gespräch.

Die Teilnahme an der Studie ist freiwillig und kann jederzeit ohne Angabe von Gründen durch Sie beendet werden, ohne dass Ihnen hierdurch Nachteile in Ihrer medizinischen Betreuung entstehen.

Studien sind notwendig, um verlässliche neue medizinische Forschungsergebnisse zu gewinnen. Unverzichtbare Vorraussetzung für die Durchführung einer Studie ist jedoch, dass

Sie Ihr Einverständnis zur Teilnahme an dieser Studie schriftlich erklären. Bitte lesen Sie den folgenden Text als Ergänzung zum Informationsgespräch mit Ihrem Studienarzt sorgfältig durch und zögern Sie nicht, Fragen zu stellen.

Bitte unterschreiben Sie die Einwilligungserklärung nur

- wenn Sie Art und Ablauf der Studie vollständig verstanden haben,
- wenn Sie bereit sind, der Teilnahme zuzustimmen und
- wenn Sie sich über Ihre Rechte als Studienteilnehmer im Klaren sind.

Zu dieser Studie, sowie zur Patienteninformation und Einwilligungserklärung wurde von der Ethikkommission eine befürwortende Stellungnahme abgegeben.

Hintergrund der Studie:

Die häufigsten Todesursachen in Deutschland und in der westlichen Welt stehen in unmittelbarem Zusammenhang mit Gefäßverkalkungen und Veränderungen der Gefäßfunktion. Bestimmte Risikofaktoren wie die terminale Niereninsuffizienz beschleunigen Gefäßverkalkungen und führen zu einer eingeschränkten Gefäßfunktion.

Bestimmte Bestandteile in Obst, Gemüse, Tee, Rotwein, und auch in Kakaobohnen haben einen positiven Effekt auf die Gefäßverkalkung und –funktion. In kürzlich erfolgten Studien konnten diese Bestandteile näher bestimmt und ferner gezeigt werden, dass sich nach einer Einnahme dieser Bestandteile die Gefäßfunktion verbessert. Diese Stoffe werden als Polyphenole und genauer als Flavanole bezeichnet und sind konzentriert in Kakaobohnen enthalten.

Die molekularen Mechanismen der Wirkungsweise der Flavanole sind noch nicht genau erörtert worden. Flavanole könnten einerseits freien Radikalen entgegenwirken und so die Gefäße schützen. Anderseits könnten Flavanole auch über eine Aktivitätssteigerung von Enzymen wirken, welche die Gefäßfunktion positiv beeinflussen.

Sie leiden unter einer terminalen Niereninsuffizienz. Diese führt zu einer schnell fortschreitenden Atherosklerose. Neue Behandlungsmethoden sind wichtig um das sehr

rasche Fortschreiten der Gefäßverkalkung aufzuhalten und somit Herzinfarkten und Schlaganfällen vorzubeugen.

Aufbau der Studie:

Wenn Sie sich dazu entschließen, an unserer Studie Teilzunehmen, würden Sie einer Kurzzeit- und/oder Langzeitstudie zugeordnet werden.

Bei der Kurzzeitstudie werden die akuten Effekte des flavanolreichen Getränkes ermittelt, dabei werden die weiter unten beschriebenen Untersuchungen an einem Tag wiederholt durchgeführt. Um eine Unverfäschltheit der Ergebnisse zu garantieren werden Sie an 2 gleichen Wochentagen untersucht werden, mit einer sich abwechselnden Dosis an Flavanolen im einzunehmenden Getränk. Die Untersuchungen finden an dialysefreien Tagen statt. Die Zuordnung zu der jeweiligen Gruppe/Dosis erfolgt randomisiert, d. h. nach dem Zufallsprinzip. Hintereinander werden Sie somit eine abwechselnde Dosis des flavanolhaltigen Getränkes erhalten. Die Reihenfolge der flavanolhaltigen Getränke, ob zuerst das flavanolreiche und dann das flavanolarme Getränk eingenommen werden soll, oder genau umgekehrt, ist ebenfalls randomisiert, also nach dem Zufallsprinzip.

In der Langzeitstudie sollen die langfristigen Effekte der Flavanole auf die Gefäße bestimmt werden. Sie würden einer Vergleichsgruppe zugeordnet werden und somit entweder eine schon ermittelte hohe Dosis an wirksamen Flavanolen einnehmen, oder eine ganz niedrige Dosis, welche keine Wirkung erzielt. Die Zuordnung zu der jeweiligen Dosis erfolgt randomisiert, d. h. nach dem Zufallsprinzip. Weder wir noch Sie wissen und können beeinflussen, welcher Gruppe/Dosis Sie zugeordnet werden. In jeder Gruppe werden gleichviele Teilnehmer untersucht werden. Dieses Getränk, welches Sie zweimal täglich einnehmen müssen, erhalten Sie über die Dauer von 30 Tagen. Am ersten und letzten Studientag werden unten beschriebene Untersuchungen durchgeführt. Die Untersuchungen finden an den gewohnten Dialysetagen statt. Einmal pro Woche kann ein Telefonat erfolgen um das Befinden nach regelmäßiger Einnahme des Studiengetränkes zu erfragen.

Welche Untersuchungen werden durchgeführt?

Bei Ihnen werden nichtinvasive (ohne Eingriffe in den Körper) Untersuchungen der Gefäße mittels Ultraschall durchgeführt werden. Ultraschalluntersuchungen gehen nicht mit Strahlenbelastung einher und es sind ferner keine Nebenwirkungen bekannt.

Bei der Untersuchungsmethode der Fluss-mediierten-Dilatation (FMD) soll die Weitstellung der Gefäße und somit die Verbesserung der Gefäßfunktion durch dargestellt werden. Dazu wird zunächst unter Ruhebedingungen ein Bild des Oberarmgefäßes aufgezeichnet, dann wird für 5 Minuten eine Blutdruckmanschette am Unterarm aufgeblasen und im Anschluss, nach Ablassen der Stauung, weitere Ultraschallbilder aufgenommen. Nach einer kleinen Pause und bei ausreichend hohen Blutdruckwerten wird Ihnen ein Medikament (Nitrolingual) verabreicht, um den Effekt einer zusätzlichen Weitstellung der Gefäße zu dokumentieren.

Durch den Arteriograph kann mittles nichtinvasiver Technik die Gefäßelastizität und die arterielle Gefäßsteifigkeit dargestellt werden. Dazu werden Messungen in Ruhe und nach Aufblasen und Ablassen einer Blutdruckmanschette am Oberarm durchgeführt.

Letztendlich werden Blutuntersuchungen durchgeführt. Hierfür werden Ihnen etwa 30 ml Blut entnommen. Aus diesen Blutproben werden einerseits die Konzentration der Flavanole und die Stoffwechselprodukte derselben und anderseits Ihre Nierenwerte, Blutfettwerte, Elektrolyte, Blutbild, Eisenhaushalt, Entzündungswerte und Urinstatus bestimmt.

Sind Nebenwirkungen bekannt oder zu erwarten?

Durch die Blutabnahme kann es zum Entstehen blauer Flecken im bereich der Einstichstelle kommen. Es besteht das sehr geringe Risiko einer lokalen oder allgemeinen Infektion. In Extrem seltenen Fällen kann es zu einer Verletzung eines Hautnerven, evtl. sogar mit chronischem Verlauf, kommen.

Die Untersuchungsmethoden der Fluss-mediierte-Dilatation (FMD) und der Gefäßsteifigkeitsmessung sind bisher von allen Patienten sehr gut vertagen worden.

Das bei der FMD verabreichte Nitrolingual wird Ihnen in niedriger Dosierung gegeben (0,4 mg Nitroglyzerin Zerbeißkapsel) und wird benötigt um eine endothelunabhängige Dilatation der Gefäße zu dokumentieren, das bedeutet eine körperfremde Antwort der Gefäße auf einen externen Stimulus. Die Halbwertzeit des Medikamentes ist im Minutenbereich. Selten

können hierdurch potentiellen Nebenwirkungen, für ca. 30 Sekunden bis maximal 5 Minuten, wie transiente Kopfschmerzen, Hitzewallungen und Blutdruckabfälle in hohen Dosierungen auftreten. Beinhochlagerung sowie Volumengabe sind die adäquate Therapie bis zum Abklingen der Wirkung. Der Blutdruck wird diesbezüglich regelmäßig kontrolliert werden. Sollte eine Unverträglichkeit oder Kontraindikation gegen Nitrolingual bestehen, werden Sie dieses Medikament natürlich nicht bekommen.

Auswirkungen auf den Elektrolythaushalt (Kalium- und Phosphat) sind nicht zu erwarten, da das verabreichte Getränk eine sehr geringe Kalium und Phosphatdosis beinhaltet (Inhaltsstoffe siehe Anhang) und Sie somit das Getränk in Ihren tägliche Speiseplan integrieren können. Vor Abschluss der jeweiligen Studientage werden jedoch zusätzlich Elektrolytwerte sowie der Blut-pH wert bestimmt werden.

Die Teilnahme an der Studie und die Einnahme des Getränkes wird Ihr Dialyse-Verhalten nicht beeinflussen, so dass Sie an gewohnten Tagen dialysieren können.

Versicherung und Obliegenheiten

Es wird für Sie eine Patientenversicherung abgeschlossen. Versicherer ist die HDI-Gerling Versicherung AG (Probandenversicherung-Nr.: 65 958420 03028, Am Schönenkamp 45, 40599 Düsseldorf, Tel: 0211-74825404, Fax: 0211-7482465). Für die dialysefreien Untersuchungstage wird zusätzlich eine Wegeunfallversicherung abgeschlossen. Versicherer ist die SV SparkassenVersicherung AG (Versicherungsnummer: 50 034 076/ 700, Bahnhofstrasse 69, 65185 Wiesbaden, Tel: 0611-1782531, Fax: 0611-1782877).

Folgende Obliegenheiten müssen eingehalten werden, ansonsten erlischt der Versicherungsschutz: Nach einem Unfall, der voraussichtlich eine Leistungspflicht herbeiführt, müssen Sie als versicherte Person unverzüglich einen Arzt hinzuziehen, seine Anordnungen befolgen und uns unterrichten. Die von der Versicherung übersandte Unfallanzeige muss wahrheitsgemäß ausgefüllt werden. Darüber hinaus geforderte sachdienliche Auskünfte müssen in gleicher Weise erteilt werden. Werden Ärzte durch die Versicherung beauftragt, müssen Sie sich auch von diesen untersuchen lassen. Die notwendigen Kosten einschließlich eines dadurch entstandenen Verdienstausfalls trägt die Versicherung. Die Ärzte, die Sie - auch aus anderen Anlässen - behandelt oder untersucht

haben, andere Versicherer, Versicherungsträger und Behörden sind zu ermächtigen, alle erforderlichen Auskünfte zu erteilen. Hat ein Unfall den Tod zur Folge, ist dies innerhalb von 48 Stunden der Versicherung zu melden, auch wenn der Unfall schon angezeigt war. Der Versicherung ist das Recht zu verschaffen, gegebenenfalls eine Obduktion durch einen von dieser beauftragten Arzt vornehmen zu lassen.

Was tun bei Auftreten von Beschwerden und/oder Begleiterscheinungen?

Sollten im Verlauf der Studie irgendwelche Beschwerden oder Begleiterscheinungen auftreten müssen Sie diese umgehend dem zuständigen Arzt mitteilen, bei schweren Begleiterscheinungen umgehend, ggf. telefonisch (Telefonnummer siehe unten). Ferner sollten Sie sich bei stärkeren Beschwerden immer an Ihren Arzt wenden oder auf der Notaufnahmestation Ma01, MNR, der Uniklinik vorstellig werden.

Wann wird die Studie vorzeitig beendet?

Sie können jederzeit, auch ohne Angabe von Gründen, Ihre Teilnahem an dieser Studie widerrufen und aus der Studie austreten, ohne dass Ihnen dadurch irgendwelche Nachteile entstehen. Ihr Studienarzt wird Sie über alle neuen Erkenntnisse, die in Bezug auf diese Studie bekannt werden und für Sie wesentlich werden können, umgehend informieren. Auf dieser Basis können Sie dann ihre Entscheidung zur weiteren Teilnahme an dieser Studie neu überdenken.

Es ist aber auch möglich, dass Ihr Studienarzt entscheidet, Ihre Teilnahme an der Studie vorzeitig zu beenden, ohne vorher ihr Einverständnis einzuholen. Die Gründe hierfür können sein:

- Sie können den Erfordernissen aus medizinischen Gründen nicht entsprechen und
- Ihr behandelnder Arzt hat den Eindruck, dass eine weitere Teilnahme an der Studie nicht in Ihrem Interesse ist.

Kontaktpersonen:
Univ.-Prof. Dr. med. T. Rassaf
Oberarzt, Hauptprüfer

Dr. med. C. Rammos
Wissenschaftlicher Mitarbeiter, Prüfarzt

Klinik für Kardiologie, Pneumologie und Angiologie
Moorenstrasse 5
40225 Düsseldorf
Tel: 0049-211-8118800
Fax: 0049-211-8118812

Inhaltsstoffe der Studiengetränke

	**Flavanolarmes Getränk**	**Flavanolreiches Getränk**
Kakao Flavanol (mg)	ND	410
Monomere (mg)	ND	62
(-)- Epicatechin (mg)	ND	55
(-)- Catechin (mg)	ND	6
Dimere (mg)	ND	62
Trimere-Decamere (mg)	ND	285
Theobromin (mg)	56	50
Koffein (mg)	5	7
Energie (kcal)	25	25
Fett (g)	0	0
Kohlenhydrate (g)	6	6
Proteine (g)	0	0
Natrium (mg)	3	2
Kalium (mg)	76	100

ND= nicht detektierbar

D: Aufwandsentschädigung

Universitätsklinikum Düsseldorf
Klinik für Kardiologie, Pneumologie und Angiologie
Direktor: Univ.- Prof. Dr. M. Kelm

Aufwandsentschädigung

Für die Teilnahme an der Studie:

"Effekte einer flavanolreichen diätischen Intervention auf die vaskuläre Funktion bei Patienten mit terminaler Niereninsuffizienz"

Der unten genannte Proband hat an der Studie teilgenommen. Und erhält als

Aufwandsentschädigung den unten aufgeführten Betrag

von 150,- Euro Akutstudie

von 250,- Euro Langzeitstudie (nichtzutreffendes streichen)

Name: Geburtsdatum:

Straße:

Wohnort:

Kontonummer: Bankleitzahl:

Unterschrift Proband Unterschrift Projektleiter

OA Prof. Dr. med. T. Rassaf

E: Inhaltsstoffe der Prüfgetränke

	Flavanolarmes Getränk	Flavanolreiches Getränk
Kakao Flavanol (mg)	ND	410
Monomere (mg)	ND	62
(-)- Epicatechin (mg)	ND	55
(-)- Catechin (mg)	ND	6
Dimere (mg)	ND	62
Trimere-Decamere (mg)	ND	285
Theobromin (mg)	56	50
Koffein (mg)	5	7
Energie (kcal)	25	25
Fett (g)	0	0
Kohlenhydrate (g)	6	6
Proteine (g)	0	0
Natrium (mg)	3	2
Kalium (mg)	76	100

ND= nicht detektierbar
