# Supplementary material for: Filtration of Macrophage Migration Inhibitory Factor (MIF) in Patients with End Stage Renal Disease Undergoing Hemodialysis
Source: PLoS One. 2015 Oct 20;10(10):e0140215. doi: 10.1371/journal.pone.0140215 (PMC4617461; doi:10.1371/journal.pone.0140215)
Supplement: S2 Protocol — (PDF) [file pone.0140215.s002.pdf]

Protocol S1: Study protocol

Patients:

The included patients (n=29) of the present trail represent a consecutively assigned sample of a larger group of patients (randomized trial) in which the influence the influence of flavanols on vascular function in ESRD patients is investigated and which has not been published yet. Serum levels of inflammatory markers (e.g. macrophage migration inhibitory factor (MIF)) were compared with those obtained from n=20 gender- and aged-matched volunteers after obtainment of written informed consent.

Inclusion criteria:

Age>18 years old, chronic kidney disease according to stadium V NKF/ KDOQI, legal capacity.

Exclusion criteria:

Exclusion criteria were age less than 18 years, anemia, hyperkalemia, active malignancies, heart failure (NYHA III-IV), severe heart rhythm disorders, pregnancy or missing save contraception (Pearl index <1%), acute infectious disease or participation in other clinical studies that might interfere with the current study.

Data collection

Baseline characteristics regarding demographic and laboratory parameters as well as clinical data were collected after assignment to the study and before, during and after hemodialysis session.

Laboratory Tests

Determination of laboratory routine parameters was performed from the Institute of Clinical Chemistry and Laboratory Diagnostic, University Hospital Duesseldorf unless noted otherwise. Markers of oxidative stress oxidized LDL (OxLDL, Mercodia, Uppsala, Sweden) were measured by ELISAs following the manufacturers protocol.

MIF ELISA

MIF was determined as described previously.(1, 2) Heparinized full blood was centrifuged at 800 g for 10 min (4° Celsius). The resulting plasma aliquots were snap frozen in liquid nitrogen and stored at -80° Celsius until further analysis.

For the determination of MIF in the dialysate ultrafiltrate samples were taken from the Genius dialysis system 30 minutes after initiation and 30 minutes before end of the HD-session, respectively.

MIF levels were measured by quantitative sandwich enzyme-linked immunosorbent assay (ELISA) (Quantikine, R&D Systems, Minneapolis, USA) according to the manufacturer's protocols.

References

1. Sobierajski J, Hendgen-Cotta UB, Luedike P, Stock P, Rammos C, Meyer C, et al. Assessment of macrophage migration inhibitory factor in humans: protocol for accurate and reproducible levels. Free Radic Biol Med. 2013;63:236-42.
2. Rammos C, Hendgen-Cotta UB, Pohl J, Totzeck M, Luedike P, Schulze VT, et al. Modulation of circulating macrophage migration inhibitory factor in the elderly. BioMed research international. 2014;2014:582586.
